# An expandable embryonic stem cell-derived Purkinje neuron progenitor population that exhibits in vivo maturation in the adult mouse cerebellum

**DOI:** 10.1038/s41598-017-09348-1

**Published:** 2017-08-18

**Authors:** Gustavo A. Higuera, Grazia Iaffaldano, Meiwand Bedar, Guy Shpak, Robin Broersen, Shashini T. Munshi, Catherine Dupont, Joost Gribnau, Femke M. S. de Vrij, Steven A. Kushner, Chris I. De Zeeuw

**Affiliations:** 1 Department of Neuroscience, Erasmus MC Rotterdam, NL-3015 GE Rotterdam, The Netherlands; 2 The Netherlands Institute for Neuroscience, Royal Netherlands Academy of Arts and Sciences, 1105 BA Amsterdam, The Netherlands; 3 Department of Psychiatry, Erasmus MC Rotterdam, NL-3015 GE Rotterdam, The Netherlands; 4 Department of Endocrinology & Reproduction, Erasmus MC Rotterdam, NL-3015 GE Rotterdam, The Netherlands

## Abstract

The directed differentiation of patient-derived induced pluripotent stem cells into cell-type specific neurons has inspired the development of therapeutic discovery for neurodegenerative diseases. Many forms of ataxia result from degeneration of cerebellar Purkinje cells, but thus far it has not been possible to efficiently generate Purkinje neuron (PN) progenitors from human or mouse pluripotent stem cells, let alone to develop a methodology for *in vivo* transplantation in the adult cerebellum. Here, we present a protocol to obtain an expandable population of cerebellar neuron progenitors from mouse embryonic stem cells. Our protocol is characterized by applying factors that promote proliferation of cerebellar progenitors. Cerebellar progenitors isolated in culture from cell aggregates contained a stable subpopulation of PN progenitors that could be expanded for up to 6 passages. When transplanted into the adult cerebellum of either wild-type mice or a strain lacking Purkinje cells (*L7cre-ERCC1* knockout), GFP-labeled progenitors differentiated *in vivo* to establish a population of calbindin-positive cells in the molecular layer with dendritic trees typical of mature PNs. We conclude that this protocol may be useful for the generation and maturation of PNs, highlighting the potential for development of a regenerative medicine approach to the treatment of cerebellar neurodegenerative diseases.

## Introduction

Purkinje neurons (PNs) are the sole output neurons of the cerebellar cortex^1^. Degeneration of PNs causes severe motor coordination deficits, referred to as ataxia^2, 3^. Cell therapy aimed at replacing diseased Purkinje neurons represent a potential cure for this type of disorder. Donor cells used in the first cerebellar transplantation studies were Purkinje progenitor cells obtained from the embryonic cerebellum^4–6^. While designing a therapeutic strategy in mouse models, cerebellar scientists tried to take advantage of the molecular and cellular mechanisms uncovered during their developmental studies^7–9^. For example, during the final maturation phase, PNs were found to develop extensive dendrites with spines that receive synaptic inputs from granule cell axons, which exert a trophic effect through glutamate release and subsequent calcium influx^10, 11^. In addition, Bergmann glia cells were found to contribute to the development and maturation of PNs by promoting their migration and glutamate homeostasis^12^. Thus, in order to derive PNs with a normal dendritic arborisation in culture, cerebellar dissociated primary cell cultures were prepared from postnatal cerebella^13–16^. Importantly, when such isolated primary progenitors were injected into the cerebellum of embryonic or young postnatal mice, the PNs were able to functionally integrate in their surrounding neuropil and receive active synaptic input^15, 16^. However, the capacity of grafted cerebellar progenitors to properly integrate into the recipient circuitry diminishes as the development of the host advances^17^.

Over the past decade, the development of differentiation protocols from pluripotent stem cells has led to the advancement of *in vitro* generation of neurons^18^, including those of the cerebellum^19–22^. Potentially, these technical advances might be useful for further developing treatments for degenerative forms of ataxia as they permit use of genetically homologous patient-derived cells, avoiding the rejection issue^23^. Earlier work has shown that functional PNs can be derived from human ES cells, and that these exhibit substantial self-organizing potential for generating a polarized structure reminiscent of the early human cerebellum at the first trimester^19, 22^. In addition, PN progenitors from mouse ES cells migrate to the Purkinje cell plate with their axons approaching the cerebellar nuclei in hosts up to E16^20^. But successful maturation and integration of ES cell-derived cerebellar progenitors has not been reported in adult recipients, which present a more challenging environment for grafted cells^17^. Moreover, until now standardization of differentiation protocols of neural progenitor cells (NPCs) has not led to consistent and robust generation of cerebellar neurons from transgenic mouse models and/or human patients with cerebellar disorders. To date, it has remained unclear what is the best strategy to consistently mature PNs derived from pluripotent stem cells at high numbers *in vitro*, allowing subsequent functional implantation and integration in adult animals *in vivo*.

Here, we generated PN progenitors from mouse ES cells implementing a cerebellar differentiation protocol of embryoid bodies (EBs) using insulin, FGF2 and cyclopamine so as to fine-regulate the balance between cerebellar neurons and glia cells, while evaluating the expression of cell-specific factors like Engrailed 2 (En2) and Neph3^24^. We subsequently promoted their proliferation and expansion by using an NPC medium containing FGF2, B27 and N2 on a substrate of the neuroprotectant laminin^25^. In addition, we facilitated their maturation *in vitro* in NS21 medium, which has been shown to optimize the *in vitro* micro-environment of primary neurons^26^. The maturation potential of these NPCs was tested *in vivo* in mice with or without host PNs^27^, using a prematurely aging mouse model characterized by neuronal degeneration, inflammation and behavioural disorders. We show that our protocol allows for the generation of an expandable PN progenitor population that can be matured both *in vitro* and *in vivo*, paving the way for future studies that focus on the integration of ES cell derived PNs into the intact adult mouse cerebellum.

## Results

**Figure 1:**
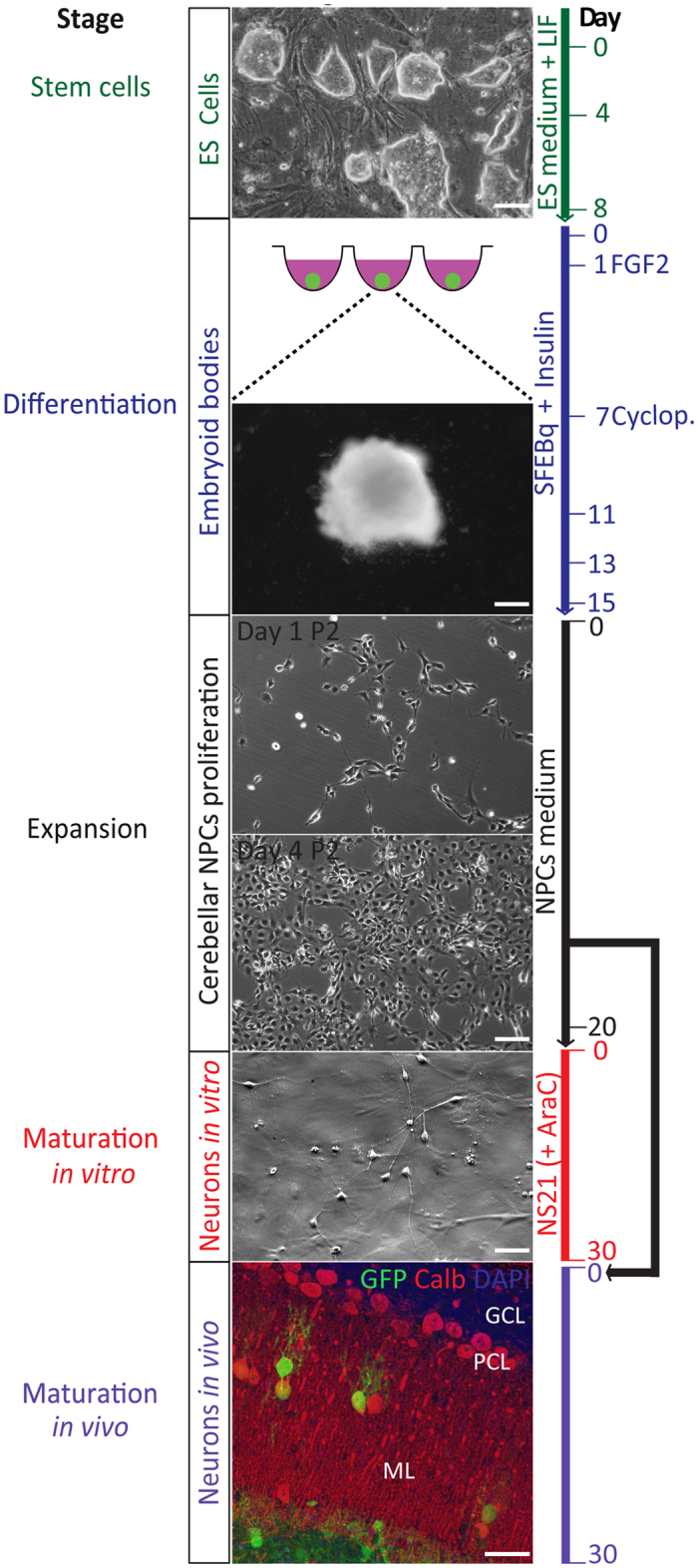
Timeline (from top to bottom) for neuronal differentiation of mouse embryonic stem cells (ES cells) into an expandable population of cerebellar neurons. Graphs show the stem cell stage (top panel: ES stage), the induction stage of embryoid bodies (EB) in SFEBq culture (EB differentiation stage), an intermediate expansion or proliferation stage of neural progenitor cells (NPCs) through stable passaging on laminin in NPCs medium (NPC expansion stage), and finally the maturation *in vitro* in NS21 medium (*in vitro* maturation stage) and maturation in adult wild type mouse cerebella (bottom panel: *in vivo* maturation stage). P2 indicates passage 2 (of expansion stage); Arac indicates cytosine β-D-arabinofuranoside; Scale bars indicate 100 μm.

We adapted existing stem cell-based cerebellar differentiation protocols^20, 28, 29^ so as to obtain high numbers of cerebellar progenitors that can be implanted *in vivo* in adult animals. We chose to isolate cerebellar progenitors from EBs, because (i) the use of a cerebellar progenitor population allows for the generation of an intermediate and stable cell state^30^ and (ii) the number of PNs that can be generated directly from ES cell cultures is limited^19–22^. To this end, we: 1) maintained and expanded mouse stem cells in ES medium (referred to as stem cell stage); 2) differentiated mouse ES cells as EBs into the cerebellar lineage (referred to as differentiation stage); 3) expanded NPCs for up to 8 passages (referred to as expansion stage); and subsequently, either 4a) induced further neurogenesis of a cerebellar progenitor population *in vitro* (referred to as *in vitro* maturation stage), or 4b) implemented integration of an expandable PN progenitor population *in vivo* (referred to as *in vivo* maturation stage) (for overview see Fig. 1).

### Stem cell stage: Generation and maintenance of ES cells

Mouse ES cells were collected and isolated as previously described^31^. Mouse ES cells were cultured and maintained^28^, while special attention was paid to obtain sharply delineated ES colonies and an appropriate ES cell density (Fig. 1).

### Differentiation stage: Cerebellar differentiation of EBs

**Figure 2:**
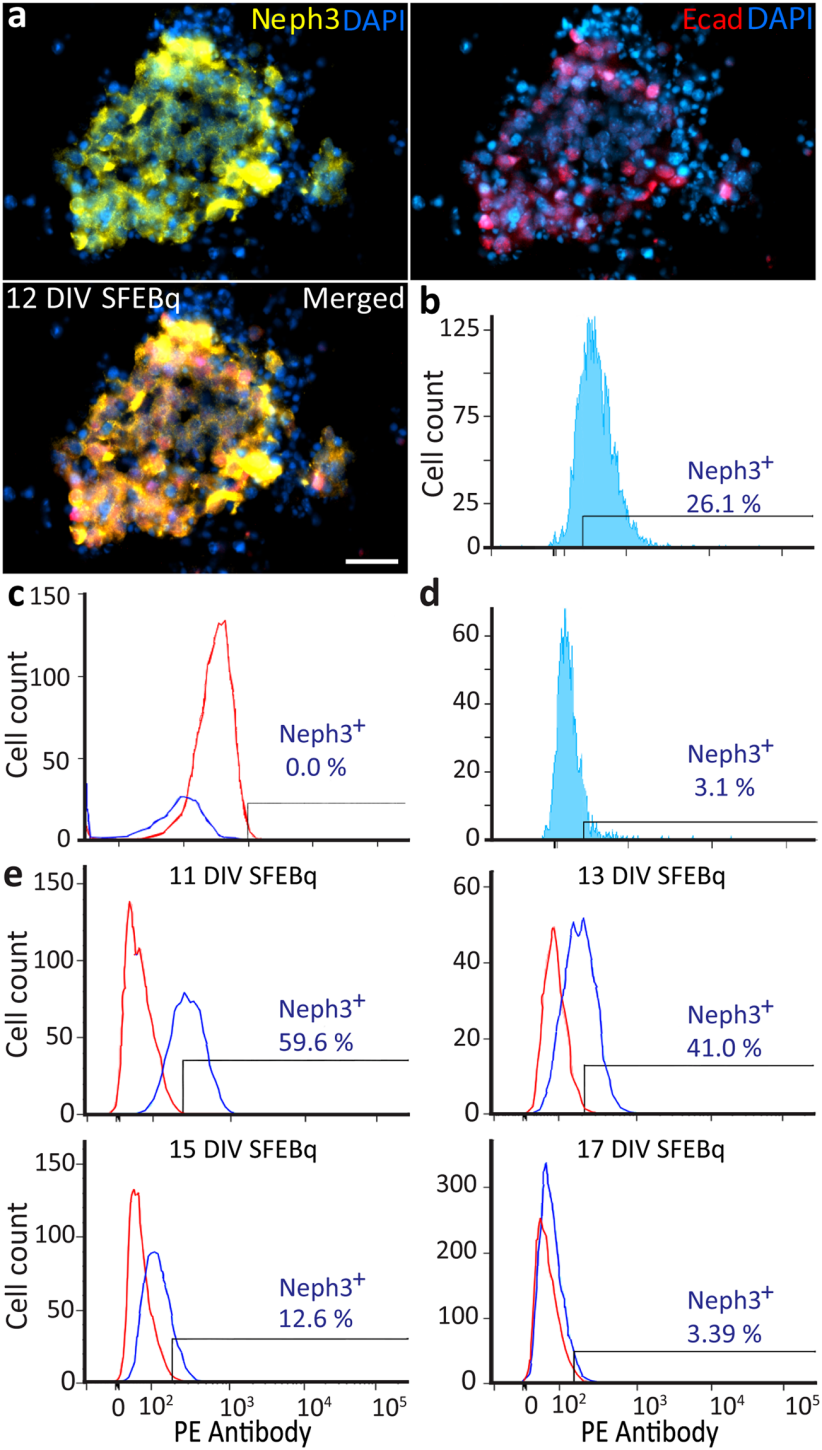
*In vitro* replication of the cerebellar anlage at the EBs induction stage (i.e. Differentiation stage). (**a**) Expression of PN progenitor markers Neph3 and E-cadherin in neuro-epithelial rosettes derived from EBs in SFEBq culture on day 12. Scale bars indicate 30 μm. (**b**) Percentage of Neph3-positive cells as determined by FACS of actin-GFP-positive cells for 13 DIV in SFEBq culture. (**c**) The percentage of Neph3-positive cells as assessed by FACS of EBs of wild type mice was negligible under static conditions without cyclopamine. (**d**) The percentage of Neph3-positive cells by FACS of actin-GFP-positive cells was very low under dynamic conditions with cyclopamine. (**e**) Percentage of Neph3-positive cells as assessed by FACS of EBs of wild type mice in SFEBq culture consistently decreased from 11 to 17 DIV (top to bottom) under static conditions with cyclopamine. The blue and red peaks reflect Neph3-positive and Neph3-negative populations, respectively.

Midbrain hindbrain boundary (MHB) patterning was induced by adding insulin, FGF2 and cyclopamine to ES cells in serum-free floating culture of embryoid body-like aggregates with quick reaggregation^20^ (SFEBq) (Supplementary Figure 1). After 8 days *in vitro* (DIV) this induction was confirmed with immunodetection of the MHB marker En2 in EBs, confirming the cerebellar fate of these cells^32, 33^. Since cerebellar differentiation may be sensitive to the size of EBs^20, 21^, we compared En2 expression under various conditions in terms of ES cell density (3 × 10^3^ or 3 × 10^4^ cells/well or 4 × 10^6^ cells/dish) and static or dynamic culture, which are all known to affect the size of EBs^34^. We used the expression of En2, Neph3 and E-cadherin, the latter two of which as markers for cerebellar ventricular zone progenitors^24^, so as to be able to determine the specific culture conditions that led to cerebellar patterning in EBs preparations. Under static conditions, En2, Neph3 and E-cadherin could be readily and homogenously induced in multiple cells contained within EB preparations at a starting population of 3 × 10^3^ cells/well (Supplementary Figure 1; Fig. 2a). Under dynamic culture conditions or at higher cell densities, the induction of the expression of these proteins was far less prominent and often limited to clumps of cells randomly distributed within EBs (Supplementary Figure 1). We also used a GFP-Actin^35^ ES cell line for fluorescence activated cell sorting (FACS) so as to closely reproduce the results of the GAD67-GFP ES cell line that has been used in the past for cerebellar mouse differentiation studies^20^. When the neural progenitors of the neuro-epithelial rosettes of EBs (with 3,000 cells per well under static conditions) were sorted for Neph3 by FACS, 26.1% of the cells were Neph3-positive (Fig. 2b). Moreover, when we tested the percentage of Neph3-positive cells under static conditions (0 RPM) without cyclopamine for control, we found that none of the cells was Neph3-positive (Fig. 2c), highlighting the impact of cyclopamine-dependent Sonic hedgehog (Shh) inhibition on differentiation of ES cells into MHB tissue^36^. In contrast, the progenitors that were Neph3-positive comprised only 3.1% of the total under dynamic conditions at 100 RPM with 3,000 cells per well with cyclopamine (Fig. 2d). It should be noted that the 26.1% of the Neph3-positive actin-GFP cells were measured at 13 DIV and that this percentage decreased over time. Indeed, when quantified by FACS in wild type cells, the percentage of Neph3-positive progenitor cells gradually decreased from 59.6% at 11 DIV, to 41.0% at 13 DIV, to 12.6% at 15 DIV and to 3.4% at 17 DIV (Fig. 2e). We tested Neph3 expression in both wild-type cells and actin-GFP cells, because the FACS data obtained by we and others^20^ indicate that it is technically easier to discriminate between Neph3-positive and Neph3-negative labeling with wild-type cells than with GFP-expressing cells (for wild-type cells see Fig. 2c and e; for actin-GFP cells see Fig. 2b and d). Indeed, the peaks of light scatter between Neph3-negative cells and Neph3-positive cells in cell lines labelled with GFP often show significant overlap.

### Expansion stage: proliferation of NPCs

**Figure 3:**
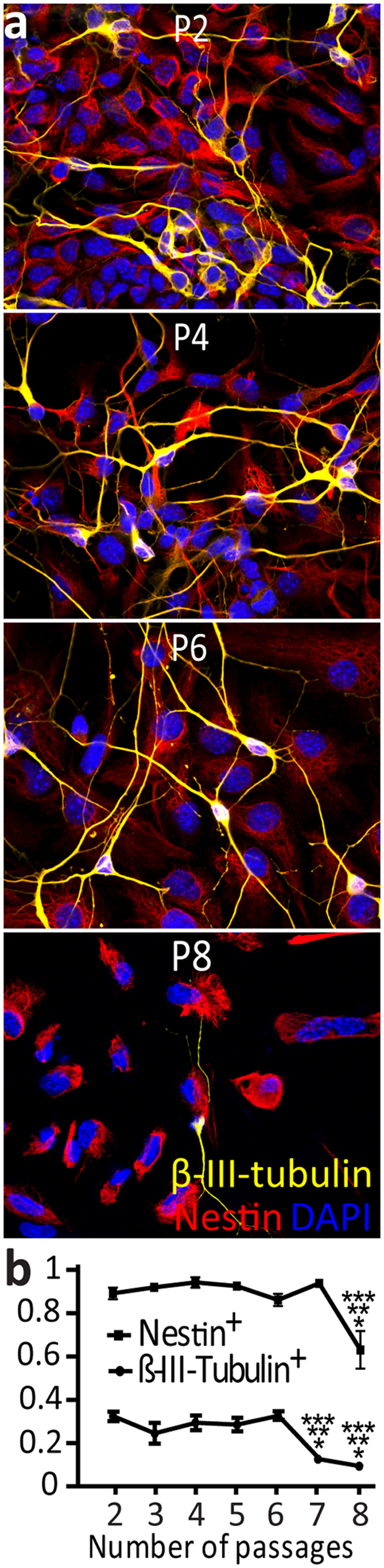
*In vitro* replication of cerebellar anlage at the expansion stage. ES cell-derived cerebellar NPCs can be expanded in NPC medium. (**a**) Expression of nestin (labeling neurons plus astrocytes) and β-III-tubulin (only neurons) in NPCs from passages (p) 2–8. Scale bar indicates 20 μm. (**b**) Quantification of the percentage of nestin-positive or β-III-tubulin-positive NPCs of the total number of cells as indicated by DAPI-staining for passage 2–8 (averaged across 10 images of three experimental runs for each passage). Note that the percentage of NPCs drops after passage 6. Error bars represent SEM. Asterisks indicate significance levels comparing the average of a passage between all passages via one-way ANOVA followed by Tukey’s with statistical significance set at P ≤ 0.01: *P < 0.01, **P < 0.001, ***P < 0.0001.

After having the SFEBq culture for 11 to 15 days in differentiation medium, we transferred the EBs to NPC medium^29^ on 10 cm^2^ 6-well plates with laminin-coating so as to promote attachment of the EBs (Fig. 3). In this proliferative stage, the cell population could be expanded and passaged up to eight times (P8) with preservation of NPCs, indicated by positive labeling for progenitor markers Nestin and β-tubulin III^37, 38^ (Fig. 3a). The β-III-tubulin-positive population significantly decreased after 7 passages (P = 2.17 × 10^−8^, from P2 to P6: 29.96 ± 1.39% of total cells as normalized to DAPI), while that for Nestin-positive significantly decreased after 8 passages (P = 1.26 × 10^−6^, from P2 to P7: 90.25 ± 0.89%) (Fig. 3b). Consequently, passaging and preservation of NPCs was performed for up to 6 passages.

Another approach involved directly sorting Neph3-positive cells with FACS from EBs and subsequently transferring them to wells. However, this did not result in stable, viable cultures of Neph3-positive progenitors (data not shown). Likewise, EBs cultured in NPC medium on plates without laminin-coating or EBs cultured in neuron-specific media, such as NS21 or DMEM/F12/FCS/N2/D-Glucose^20^, did not result in sufficient attachment of the EBs, and as a consequence did not yield viable cultures. Finally, when we tried to culture EBs that were subjected to a differentiation stage shorter than 11 DIV or longer than 15 DIV, the attachment to the laminin-coated 6-well plate surface was also insufficient to achieve prominent attachment or expansion.

### Maturation *in vitro* stage: maintenance of functional neurons in NS21 medium

**Figure 4:**
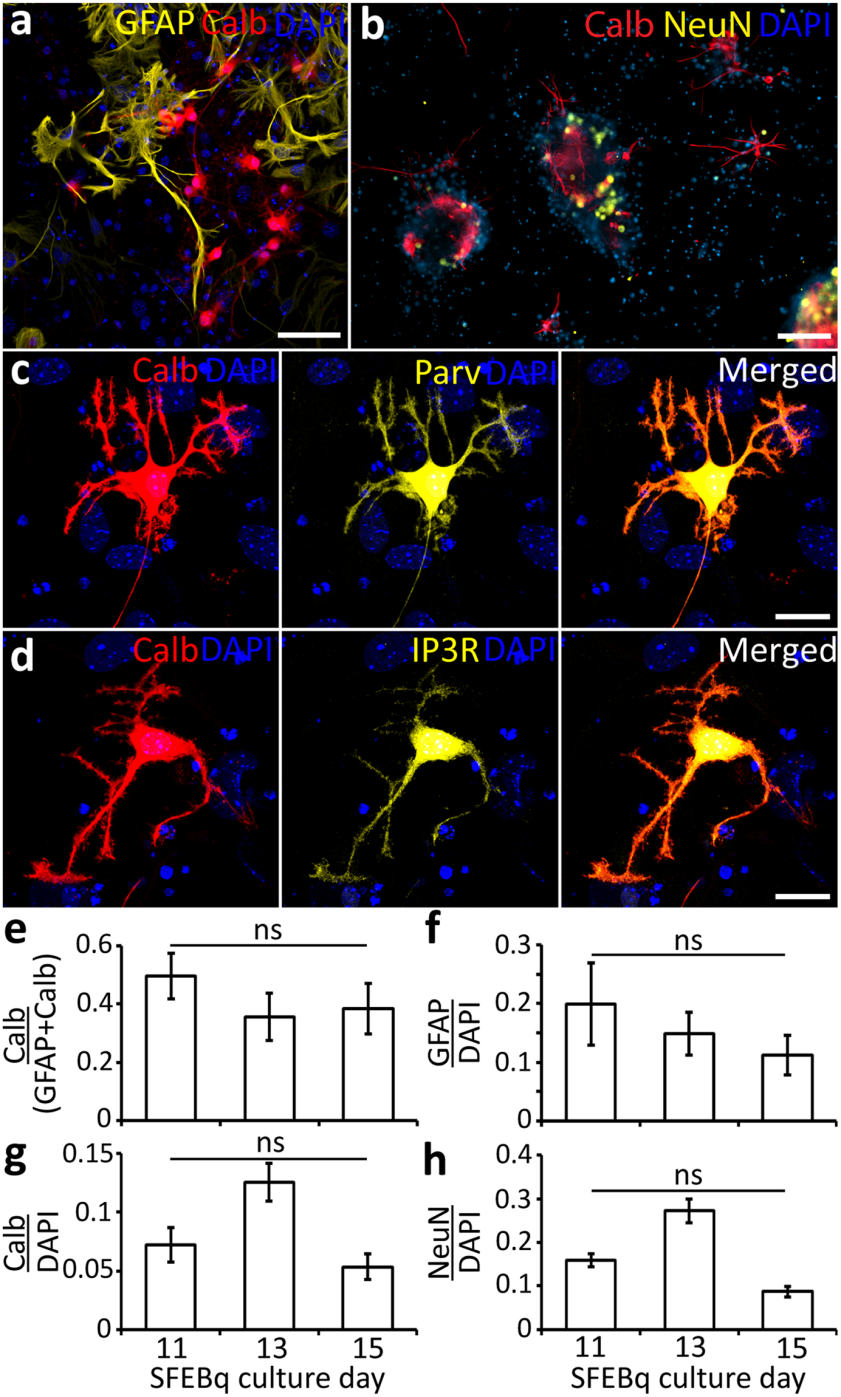
*In vitro* replication of cerebellar anlage at the maturation stage. NPCs cultured in NS21 medium matured to calbindin-positive PNs. (**a**) Culture of NPCs in NS21 medium comprised calbindin-positive and GFAP-positive cells. (**b**) Calbindin-positive and NeuN-positive cells were present after 30 DIV in NS21 medium. There was co-expression of (**c**) calbindin and parvalbumin, and (**d**) calbindin and IP3R in NPCs cultured in NS21 medium. NPCs from 11 to 15 DIV EBs in SFEBq culture presented various cell types in culture and are shown as ratios of: (**e**) calbindin-positive cells to GFAP-positive cells; (**f**) GFAP-positive cells with respect to the total DAPI-positive cells; (**g**) calbindin-positive cells with respect to the total DAPI-positive cells; and (**h**) NeuN-positive cells to the total DAPI-positive cells. Data are represented as the mean and SEM (n = 10 images from 6 experimental runs consisting of 6 cell batches), which were assessed via one-way ANOVA followed by Tukey’s with statistical significance set at P ≤ 0.01. Scale bars: 60 μm (**a**); 200 μm (**b**); 20 μm (**c** and **d**).

For the maturation stage, we generally used NPCs that were expanded for 2 to 6 passages. These cells were seeded on polyornithine-laminin coated coverslips and cultured as progenitor neurons and glia cells in NS21 medium with AraC^26^. This procedure yielded a considerable number of neurons and it confirmed the standardization of the cell state. The cells expressed calbindin, NeuN or glial fibrillary acidic protein (GFAP), which indicate PNs, cerebellar interneurons and Bergmann glia cells, respectively^38–40^ (Fig. 4a,b). The calbindin-positive cells, which displayed arborized dendrites with spines typical of PNs, also expressed other late PN markers, such as those for parvalbumin and inositol 1,4,5-trisphosphate receptor (IP3R) (Fig. 4c,d). When NPCs were cultured in this manner, all cell types were randomly distributed across the coverslip (Fig. 4a–d). If the cells were cultured in DMEM/FCS/N2/D-Glucose medium^20^ rather than NS21 medium, the ratio of calbindin-positive to GFAP-positive cells was significantly lower (P = 9 × 10^−4^) (Supplementary Figure 2). Likewise, when we used our NS21 medium without the addition of AraC, which is known to prevent the astrocytes from overgrowing the neuronal population^41^, we found that astrocytes became more prominent while neurons became scarce in culture.

The ratio of the various cell types did not significantly depend on the day of the differentiation stage at which the EBs were collected. More specifically, the ratios of calbindin-positive to GFAP-positive cells (Fig. 4e), GFAP-positive cells to DAPI-positive cells (Fig. 4f), calbindin-positive cells to DAPI-positive cells (Fig. 4g), and NeuN-positive to DAPI-positive cells (Fig. 4h) were relatively stable throughout the period in the differentiation stage during which the EBs were collected (days 11 to 15). These data suggest that it is the specific type of medium used at the maturation stage, rather than the temporal course of events during the differentiation stage, that determines the ratio of cell types.

To assess the level of maturation of the NPC-derived PNs after 15 days of culturing on coverslips, we compared the extent of their dendritic arborisation with that of PNs of dissociated cerebellar cells obtained from E16–18 mouse cerebella (Supplementary Figure 3a–c). The calbindin-positive PNs derived directly from the NPCs (NPCPN) showed significantly fewer secondary dendrites (P = 2 × 10^−4^) and significantly smaller cell bodies (P = 9.9 × 10^−5^) compared to those of calbindin-positive PNs of dissociated cells (PN), whereas the number of primary dendrites was not significantly different (P = 9.3 × 10^−2^), (Supplementary Figure 3d–f). In addition, the PNs had a significantly higher number of secondary dendrites (P = 2 × 10^−4^) than in dissociated PNs in co-culture (CPNs) and also a significantly larger size of the cell bodies (P = 9.9 × 10^−5^) than in ES-cell-derived NPCs in co-culture (GFPPN). Together, these data suggest that molecular factors produced by NPCs and/or direct contacts between cells can negatively affect the dendritic arborisation and soma growth of dissociated PNs.

Interestingly, upon direct transfer of EBs to coverslips coated with poly-ornithinine/laminin in NS21 medium - that is without passaging through the expansion stage - we could also identify a population of calbindin-positive cells that showed dendritic arbors (Supplementary Figure 4). The dendrites of these cells did not show spines, but the number of primary and secondary dendrites was 3.7 ± 1.1 and 8.3 ± 2.4, respectively, which was comparable to those of NPC-derived PNs. These data indicate that PNs directly obtained from EBs have a similar dendritic arborisation potential as passaged NPCs.

**Figure 5:**
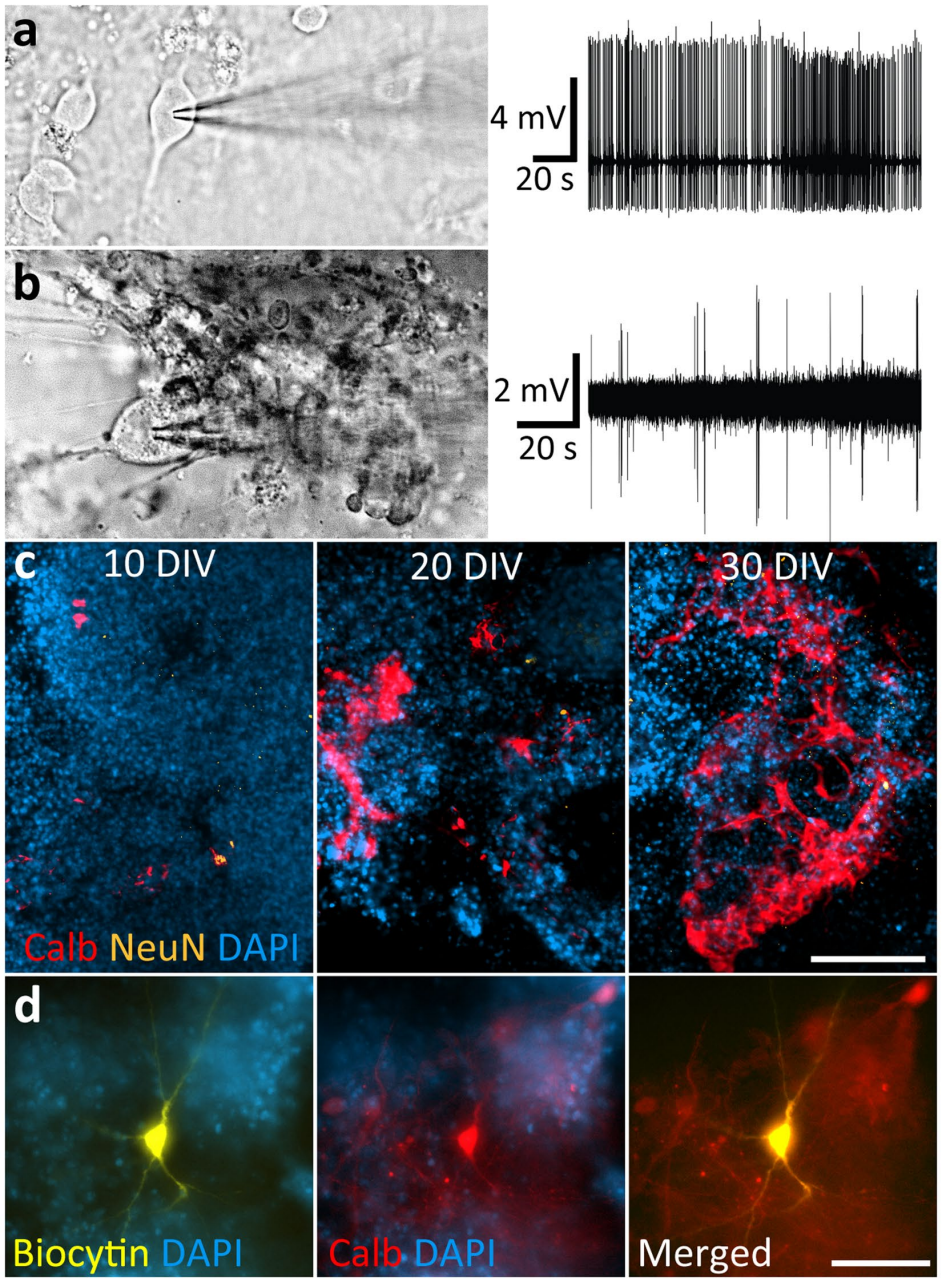
Intrinsic spiking behavior of mouse ES-cell derived PNs in the maturation stage. (**a**) NPC-derived neurons on coverslips showed intrinsic spike activity recorded in cell-attached mode (N = 21 cells). (**b**) NPCs embedded in matrigel-NS21 and deposited and cultured in µ-wells also showed intrinsic action potential spontaneous activity of neurons in cell-attached mode (N = 8 cells). (**c**) Calbindin-positive, but not NeuN-positive, cells in µ-well cultures aggregated in NS21 medium at 10 DIV, 20 DIV and 30 DIV. (**d**) A stem-cell-derived neuron in the µ-well culture that was successfully injected with biocytin following intracellular recordings (left panel) also expressed calbindin (middle panel). Scale bars: 100 µm (**c** and **d**).

Subsequently, we characterized the electrophysiological properties of PNs with *in vitro* patch-clamp recordings to determine their viability (Fig. 5a). After a maturation of 13 to 18 days in NS21 medium, NPC-derived PNs showed spontaneous intrinsic simple spike activity of 3.73 ± 1.24 Hz (n = 21 cells) and upon membrane depolarization with current injections, simple spike activity could reach a frequency of 11.1 ± 2.5 Hz. On average the mature PNs on coverslips showed an input resistance of 402 ± 30 MΩ, a resting membrane potential of −50.9 ± 2.7 mV, and a capacitance of 57 ± 6 pF. Likewise, when we cultured NPCs in 3D by depositing cells suspended in BD-Matrigel (1:2 dilution ratio)^42^ into transparent µ-wells^43^, NPC-derived PNs also showed intrinsic simple spike activity, albeit at a lower frequency (0.4 ± 0.23 Hz, n = 8 cells) (Fig. 5b). The PNs in the µ-wells showed an input resistance of 351 ± 25 MΩ, a resting membrane potential of −67 ± 3.2 mV, and a capacitance of 71.2 ± 7.8 pF. The NPC-derived PNs in the µ-wells were calbindin-positive, but not NeuN-positive, confirming the Purkinje cell identity at 10 to 30 DIV (Fig. 5c). In contrast to the PN preparation on coverslips, the 3D culture configuration allowed us to perform intracellular biocytin labeling of patched neurons with post-recording immunocytochemical confirmation of calbindin marker expression (Fig. 5d).

### Maturation *in vivo* stage: NPC-derived PNs in early post-natal and adult cerebellum

To find out whether NPC-derived PNs were suitable for *in vivo* transplantation, we injected actin-GFP-positive NPCs into the cerebellum of early postnatal or adult mice. Since integration is reportedly better during the development of the recipient cerebellum in younger animals^17, 34, 44^, where some of the developmental molecular cues relevant for the integration of progenitor PNs are still active, we first evaluated whether NPC-derived PNs would be able to migrate and mature in the recipient cerebellum of newborn mice^45^. For these transplantations, we prepared cell suspensions of NPCs (5 × 10^4^ cells/µl as measured with a Biorad® automated cell counter) in NS21 medium after 2–6 passages at the expansion stage and injected them directly into the brain without any maturation *in vitro* (i.e. without the *in vitro* maturation stage outlined above). Eight wild-type mice at postnatal day 1 received three injections of approximately 1 µl of NPCs (i.e. 5 × 10^4^ cells/µl) per injection at random depths in the vermis and/or hemispheres of their cerebellum. Thirty days after grafting, six out of the eight mice (75%) showed GFP labelling in their cerebellar cortex (Supplementary Figure 5). Only 0.17 ± 0.06% of the injected NPCs were retrieved, confirming that only a small fraction of the transplanted cells exhibit long-term survival in the postnatal cerebellum. Of the NPC-derived cells that survived grafting, 9.12 ± 1.66% showed the typical morphological presentation of maturing PNs. These cells expressed late PN markers including calbindin and parvalbumin (Supplementary Figure 5a and b). Moreover, the dendritic trees of these PNs were extensively arborized and contained spines, which received VGLUT2-positive inputs (Supplementary Figure 5c), indicating the presence of inputs from climbing fibers. Importantly, GFP-positive-calbindin-positive axon terminals were distinguishable along the soma of SMI32-positive glutamatergic neurons in the cerebellar nuclei (Supplementary Figure 5d). These data suggest that NPCs mature and can in principle form normal connections in the developing cerebellum^17, 20^.

**Figure 6:**
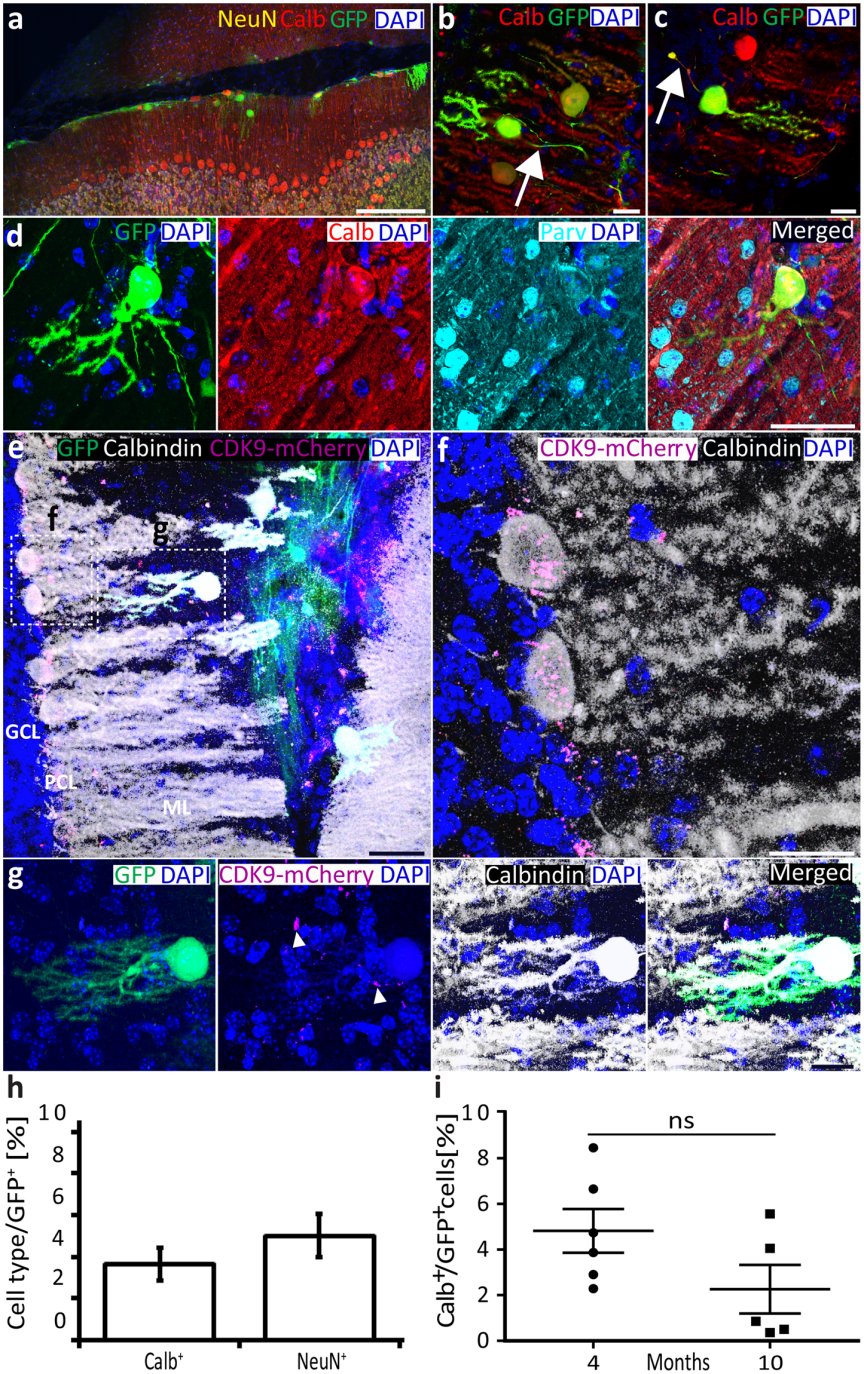
NPCs in a 10-month old adult wild type mouse cerebellum 30 days after grafting. (**a**) Integration of donor cells into the molecular layer of lobule VI (simplex). Donor cells grafted into the recipient cortex move along the pial surface. Some of them remained in the interlobular space between lobule simplex and crus 1. Other donor cells passed through the pia and integrated into the host molecular layer (inward route). (**b** and **c**) Donor GPF-positive cells matured in the Purkinje cell lineage. The bipolar morphology with the characteristic dendritic tree on the one side and the axonal process (indicated by arrows) on the opposite side indicate that the GFP-positive cells are Purkinje neurons. (**b**) Donor Purkinje cell with cell body grafted into the molecular layer shows a normal axon and dendritic tree, but an inverted orientation. (**c**) Grafted cell aligned to the host PNs exhibiting the correct orientation, but an axotomized axon. These transplanted PNs are representative of the successfully grafted donor cells. (**d**) GFP-positive-PNs co-expressed calbindin and parvalbumin. (**e**) GFP-positive NPCs grafted into an adult CDK9-mCherry mouse cerebellum showed that (**f**) only the resident PNs expressed CDK9 in their nucleus and soma. (**g**) GFP-positive donor cells expressed calbindin, but did not express CDK9-mCherry, indicating there is no fusion with resident cells. Arrowhead points to CDK9-mCherry expression inside and around nuclei of resident cells (n = 3 mice). (**h**) Quantification of calbindin-positive (n = 11 mice) and NeuN-positive (n = 16 mice) cells over a total actin-GFP-positive cells. All CDK9-mCherry micrographs were obtained from sagittal sections. (**i**) Percentage of calbindin-positive cells over a total number of actin-GFP-positive grafted cells plotted against the age of the host mouse cerebellum (n = 6 for four-month-old mice; n = 5 for ten-month-old mice). Chart shows the mean and SEM with statistical comparison assessed via one-way ANOVA followed by Tukey’s with statistical significance set at p ≤ 0.01. Scale bars: 500 μm (a); 20 μm (b, c, f and g); and 40 μm (d and e). ML: Molecular layer; PCL: Purkinje cell layer; GCL: Granule cell layer. *all micrographs represent coronal sections unless otherwise stated.

Next, to find out whether PNs can also survive and mature in an adult host, actin-GFP-positive NPCs in cell suspension (1.5 µl with 5 × 10^4^ cells/µl) were injected into the cerebellum of 16 adult wild-type mice aged 4–10 months old (Fig. 6 and Supplementary Figure 6). Thirty days after grafting, 11 out of the 16 mice (68.75%) showed GFP labelling in their cerebellar cortex (Fig. 6a). The average percentage of surviving GFP-positive cells was 2.8 ± 0.47%, which was slightly higher (P = 0.016) than that found in the early postnatal animals. In all 11 mice with surviving grafted cells, we found morphologically matured PNs, as recognized by the expression of calbindin and parvalbumin, the presence of a developed dendritic tree with spines in the molecular layer, and the morphology of axons projecting towards the white matter (Fig. 6b). Yet, some axons of the grafted PNs showed varicosities forming a terminal club (Fig. 6c), which may reflect either the failure of the axon to traverse through the white matter^46^ or a sprouting process that occurs at an infraganglionic plexus^47^.

The actin-GFP-positive cells in the adult animals comprised a population of calbindin-positive PNs (3.66 ± 0.79% of the total of actin-GFP-positive cells) (Fig. 6a–d and h) and a population of NeuN-positive cells (5.01 ± 1.03%), (Fig. 6a and h and Supplementary Figure 7). The percentage of calbindin-positive actin-GFP-positive PNs was slightly lower (P = 0.027) than that in the early postnatal animals (9.12 ± 1.66%). Whereas some of the GFP-positive cells prominently expressed calbindin in their dendritic trees (Supplementary Figure 8), others showed no or only very weak calbindin expression, despite displaying a PN-like morphology (Fig. 6b). The majority of the GFP-positive grafted PNs (92.57%) were located in the molecular layer, but some could also be detected in the Purkinje cell layer (1.01%), granular layer (6.08%), or even white matter (0.34%), presumably depending on the migratory route they took following injection into the cerebellum^48^.

Some of the grafted cells could also be situated outside the cerebellar cortical parenchyma. However, we did not observe grafted PNs or NeuN-positive neurons in the cerebellar nuclei, despite the fact that some of the injections were aimed at that locus (Supplementary Figure 6). Actin-GFP-positive cells injected into this area most probably died, as revealed by positive labelling for apoptotic marker caspase-3 (Supplementary Figure 9). Interestingly, despite its low number of surviving cells, the cerebellar cortex did not show caspase-3-positive cells, which may point towards processes such as autophagy for removal of part of the grafted progenitors^49^.

To find out to what extent NPCs matured into PNs *in vivo* due to cell fusion with host PNs nuclei, we injected GFP-NPCs in the cerebella of CDK9-mCherry-knockin mice^50^ (Fig. 6e–g). The cell-body of PNs of CDK9-mCherry-knockin mice prominently express a fluorescent form (mCherry) of CDK9, which is involved in transcription by RNA polymerase-II (Fig. 6e,f). None of the GFP-positive-PNs did express CDK9-mCherry (Fig. 6g), indicating that the NPCs maturation into PNs *in vivo* described above did not occur due to cell fusion with host PNs nuclei.

To evaluate the effect of age of the host cerebellum on NPC maturation, the percentage of calbindin-positive cells of the total actin-GFP-positive cells were compared among 4-month-old and 10-month-old mice, which included both the normal hosts and the CDK9-mCherry-knockin mice (Fig. 6i). Although the older mice (i.e. 10-month old) showed a downward trend in NPC expression of calbindin with respect to that in the younger mice (i.e. 4-month old), the difference (between 2.26 ± 1.07% and 4.81 ± 0.96% of calbindin-positive to GFP-positive cells) was not significant (P = 7 × 10^−2^).

**Figure 7:**
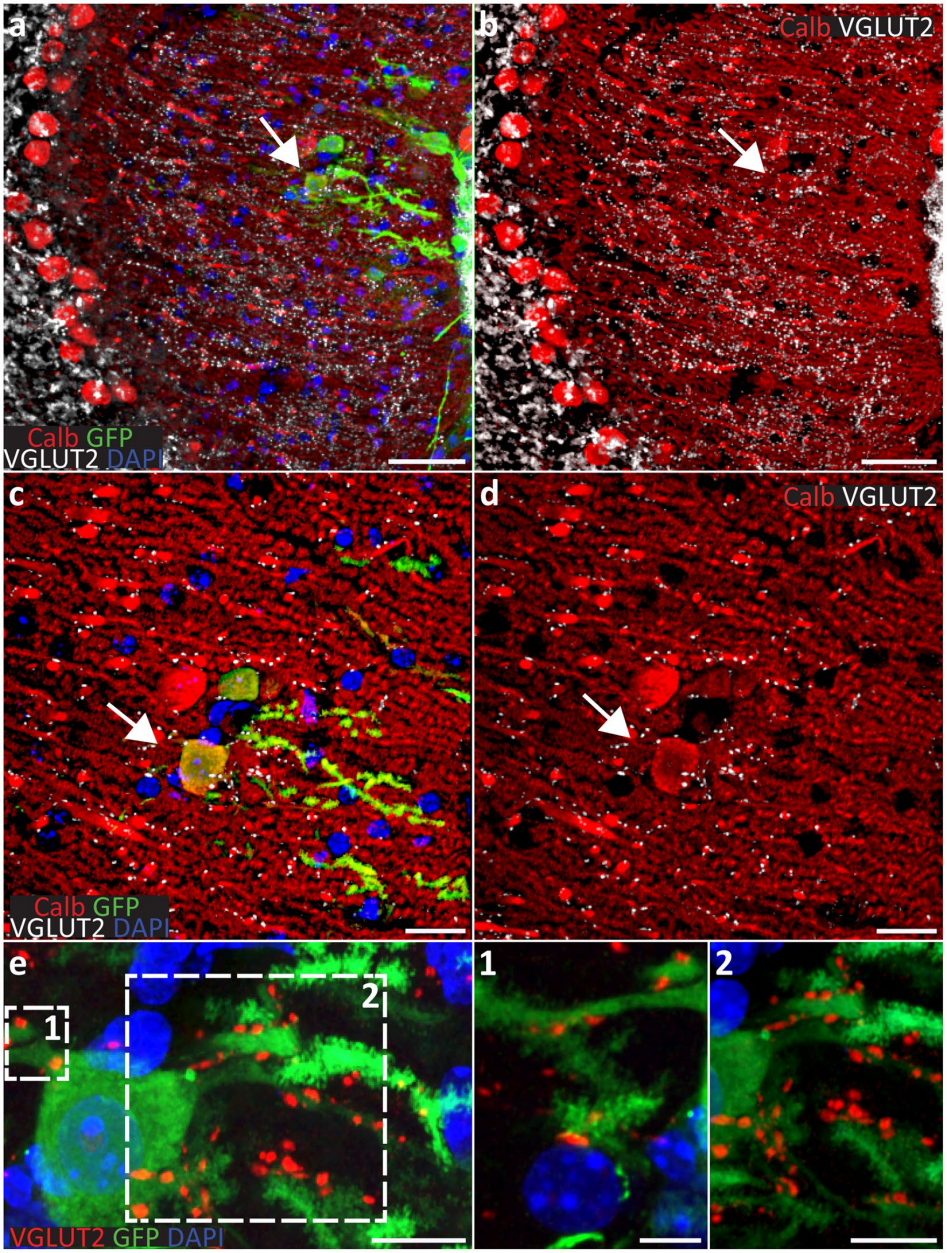
Integration into the intact molecular layer and formation of synaptic connections with afferents. (**a**) Thirty days after transplantation into the adult (10-month old) wild type cerebella some of donor GFP-positive cells (arrows) enter the intact molecular layer of lobule simplex through the pial surface. (**a**,**b**) Donor cells differentiating into Purkinje cells (**d**–**f**) adopt the PN progenitor behavior. Once the cortex is entered, they migrate along the host Bergman glia stopping at different depths of the host molecular layer. (**b**,**d**) Distribution of VGLUT2 into the host molecular layer. (**e**) Even the displaced, donor GFP-positive, calbindin-positive cells grafted into the molecular layer receive synaptic inputs from host climbing fibers as shown by the presence of the VGLUT2-positive synaptic boutons on their dendrites. Scale bars: 50 μm (**a**,**b**); 20 μm (**c**,**d**); and 10 μm (**e**). *All micrographs represent coronal sections.

**Figure 8:**
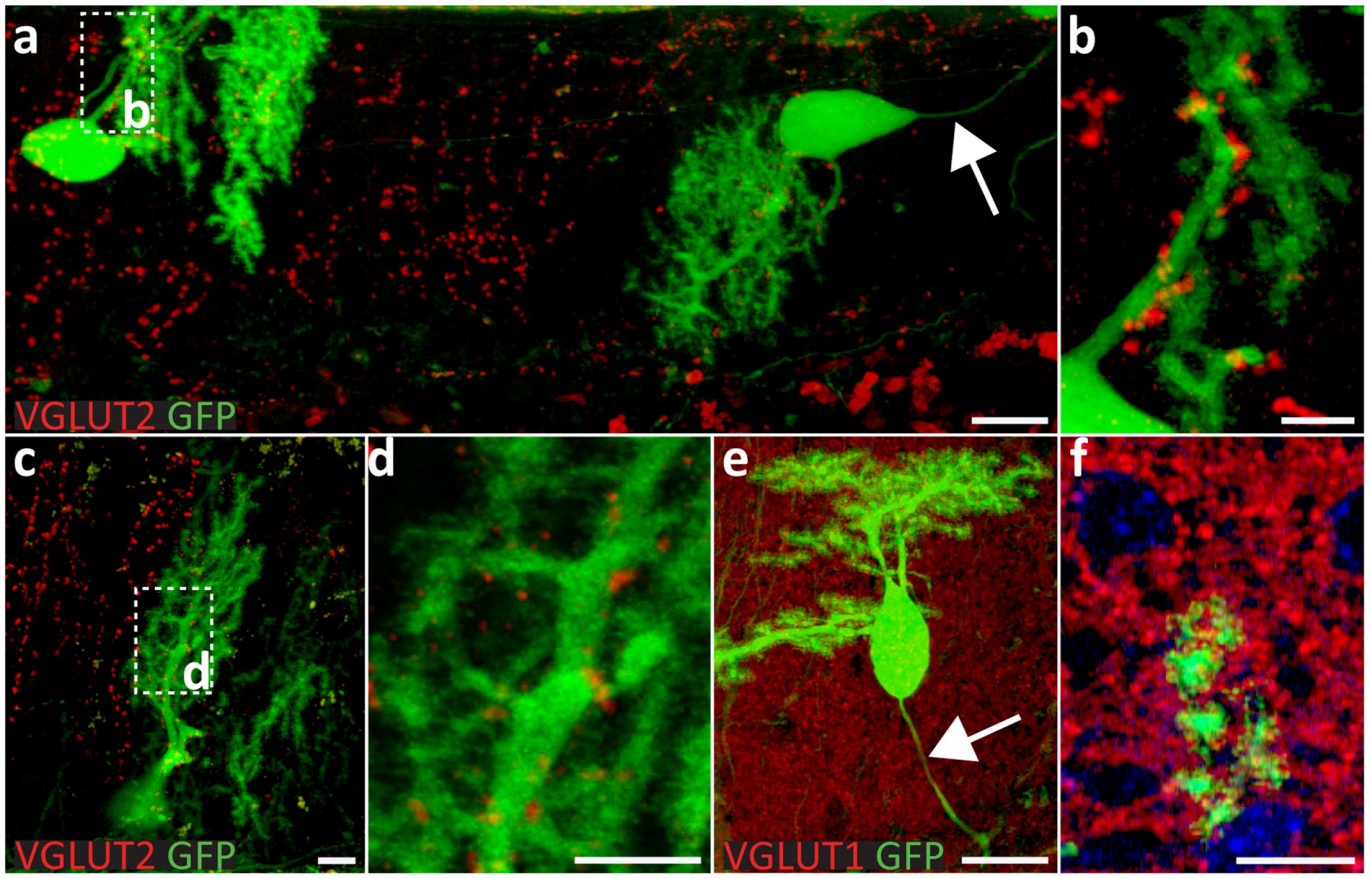
Adult (10-month old) wild type cerebella allow the synaptic integration of GFP-positive PNs in the host’s cerebellar network thirty days after transplantation to a limited extent. (**a**) Some of the donor GFP-positive cells enter the cortex in the vermis through the pial surface and settle in the host molecular layer where synaptic VGLUT2-positive boutons are located on the (**b**) proximal part of their dendrites. Arrow points to the axon of a displaced donor Purkinje cell, which is extending on the opposite side of the extensive dentritic tree. (**c**,**d**) The dendritic tree of a GFP-positive PN is enveloped by VGLUT2 positive boutons on its intermediary and distal parts. (**e**) VGLUT1-positive vesicles surround grafted GFP-positive PNs and (**f**) its distal dendritic tree. Axon is indicated by arrow. Scale bars: 10 μm (**a**,**c**,**f**); 5 μm (**b**,**d**); and 20 μm (**e**). *All micrographs represent coronal sections.

To find out whether the ES/NPC-derived grafted PNs received synaptic inputs from climbing fibers and parallel fibers in adult hosts, we investigated 30 days after grafting whether GFP-positive PNs received inputs from VGLUT2-positive and/or VGLUT1-positive boutons, respectively^51^. Both appositions were readily observed (Figs 7 and 8), confirming that the formation of synaptic inputs onto grafted PNs is possible^9, 16^. Some of the GFP-positive PNs that expressed calbindin and received input from VGLUT2-positive terminals were located in the Purkinje cell layer, but many of them were located in the molecular layer (Fig. 7a–e). However, even when grafted cells were not located in the Purkinje cell layer or not properly oriented in the molecular layer, GFP-positive PNs received VGLUT2-positive inputs at both the proximal and distal parts of their dendrites (Fig. 8a–d). Likewise, VGLUT1-positive boutons were also identified on both the proximal and distal dendrites of GFP-positive PNs (Fig. 8e,f). Interestingly, some of the VGLUT2-positive and VGLUT1-positive terminals were also apposed to the cell-bodies of GFP-negative host PNs located in the molecular layer (Fig. 7d); possibly this subcellular displacement reflects the impact of proteolytic enzymes secreted by the migrating cerebellar progenitors^48^.

**Figure 9:**
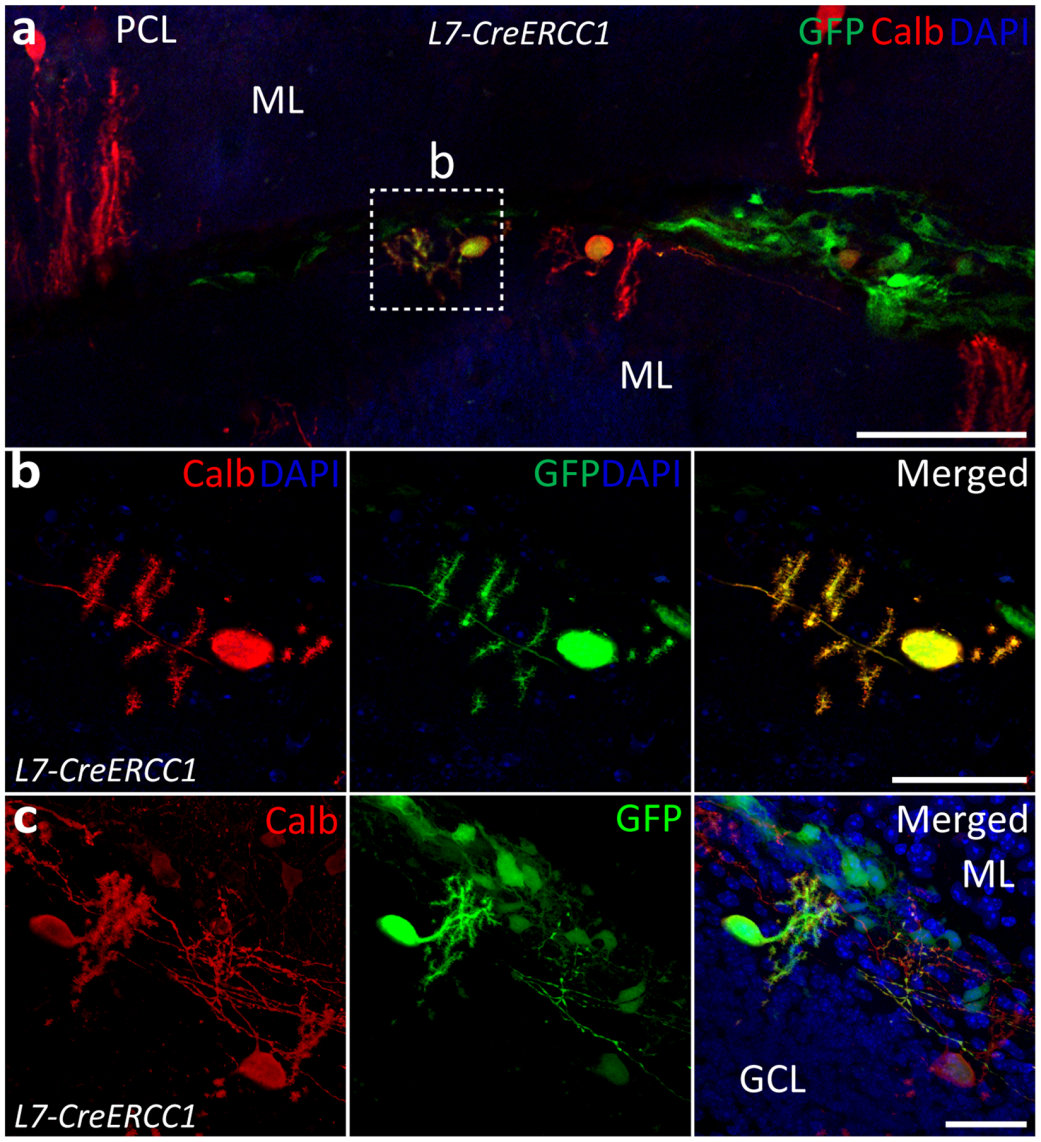
NPC injections in *L7cre-ERCC1* KO mice matured into PNs. (**a,b**) NPCs expressing actin-GFP were injected into lobules IV-V of an 8-month old mouse cerebellum and displayed maturation as shown by calbindin expression and morphological features. (**c**) Some GFP-positive PNs migrated to the approximate location where the Purkinje cell layer used to be before degeneration. All images were taken 25 days after grafting (n = 3 mice). Scale bars: 100 μm (**a**); and 40 µm (**b**,**c**). ML: Molecular layer; PCL: Purkinje cell layer; GCL: Granule cell layer. *All micrographs represent coronal sections.

Finally, to examine whether NPCs can mature in a mouse cerebellum that suffers from comprehensive resident PN death, we also injected actin-GFP-positive NPCs in the cerebellum of 8-month old *L7cre-ERCC1* KO mice, which lack resident PNs and show severe ataxia^27^ (Fig. 9). The average percentage of surviving GFP-positive cells in the *L7cre-ERCC1* KO mice was 2.48 ± 0.41%, which was comparable to wild-type animals (2.8 ± 0.47%). Additionally, 3.4 ± 0.72% of the grafted actin-GFP-positive cells also expressed calbindin, which was also consistent with the results in wild-type animals (3.66 ± 0.79%). These calbindin-positive actin-GFP-positive cells were also predominantly present in the molecular layer (Fig. 9a,b) and the layer that used to contain the host Purkinje cells (Fig. 9c). Our results indicate phenotypic similarity in the maturation of ES-cell-derived NPCs into PNs in wild type and *L7cre-ERCC1* KO mice, highlighting that our protocol allows for the maturation of PNs in the cerebellar cortex of both healthy and ataxic mice suffering from neurodegeneration.

## Discussion

In this study, we present a protocol to obtain a cerebellar neural progenitor cell population from mouse ES cells, which display maturation *in vitro* as well as in the healthy and PN deficient adult mouse cerebellum *in vivo*. The generation of stable populations of cerebellar NPCs was achieved by combining and adapting previously reported protocols for the generation of stem cell cultures^28^, neuronal progenitors^29^, and cerebellar differentiated cells^20^. By testing the cerebellar maturation potential of NPCs both *in vitro* and *in vivo*, we were able to show that the cerebellar NPCs generated in our protocol form a promising cell source for *in vivo* cerebellar transplantation. Our protocol yielded up to 59.6% PN progenitors as quantified by Neph3, which was more effective than most, if not all, other protocols published so far. For example, the amount of PN progenitors from total cultured cells as estimated by the expression of the markers L7, IP3R1-Calbindin, and Neph3 were 1 to 11%^36, 52^, 0.45%^21^, and 5 to 27.9%^19, 20, 22^, respectively in prior studies. One of the main reasons why our approach is probably more efficient is that we collect EBs from days 11 to 15 during the differentiation stage without severely decreasing the potential to later mature as PNs *in vitro or in vivo* ^53^. While PN progenitors could not be sorted, EB-derived cerebellar NPCs could be expanded and cryopreserved for up to six passages, allowing storage of large numbers of cerebellar NPCs for maturation *in vitro* and *in vivo* without having to initiate differentiation from ES cells every time. Still, the PNs obtained with our protocol also had their limitations, particularly in relation to their integration in the adult brain.

The cerebellar differentiation stage was achieved by focusing on specification of the MHB-region^19, 20, 23^. Mouse ES cells could be induced in SFEBq culture with the addition of insulin, FGF2 and cyclopamine at 0, 1 and 7 DIV, respectively^20^. Screening of engrailed-2-positive cells in SFEBq culture at 8 DIV indicated that the effectiveness of external and internal caudalizing signals not only depended on the signaling pathways themselves, but also on the size of the EBs. As EB size increased beyond 3,000 cells, engrailed-2-positive cell numbers did not increase proportionally. We expected convection to overcome the penetration problem (or diffusion limitation) of rostro-caudal and dorso-ventral patterning signals into EBs, but we found that this strategy did not result in a larger engrailed-2-positive cell population. This was probably due to the sensitivity of differentiating EBs to external cues, such as stress or dynamic culturing, which led to altered nutrient limitation, highlighting the sensitivity of cerebellar progenitors to spatial and temporal signals^54^.

The NPC expansion stage was improved by studying the expression of the PN progenitor marker Neph3, which served as an ideal marker to readily identify Purkinje progenitors in SFEBq culture^19, 20, 22, 24^. We found that Neph3 expression in neural rosettes of EBs depended on the cell line. The highest level of expression of Neph3-positive cells occurred at 11 DIV for EBs of the wild-type line vs. 13 DIV for EBs of the GFP-actin line. Since Neph3-positive cells sorted through FACS were difficult to maintain alive or keep in culture after isolation^22^, we collected cells directly from EBs for NPC culture and expansion while using Neph3 expression as a parameter to determine the optimal period. Once specification of the MHB-region was reached, neuronal and glial cell progenitor populations were born spontaneously and required no further treatment other than isolation by replating on laminin in NPC medium^29^. Laminin, which can act as a key maintenance component of the basement membrane surrounding epithelial cells during early stages of embryonic development^55^, served as an effective surface coating following neural differentiation^25, 56, 57^. FGF2, which can induce rhombomere-1 activity^58^, was also critical in that it helped NPCs to maintain their neuronal progenitor functionality, probably by keeping the population mitotically active and by preventing the initiation of terminal differentiation^59^. Our results showed that this influence of FGF2 could be sustained in NPC medium for up to 6 passages before the downregulated expression of neural progenitor markers nestin and β-III-tubulin. Thus, the NPC protocol described here yields an accessible and large cerebellar progenitor population, which allows for standardization of cerebellar differentiation^30^.

The *in vitro* cerebellar maturation of NPCs was accomplished by adjusting the culture micro-environment. Using NS21 medium^26^, we generated a suitable *in vitro* culture micro-environment for NPCs on coverslips by allowing partial maturation of intrinsically-active cerebellar neurons. The firing frequency (3.73 ± 1.24 Hz) and input resistance (402 ± 30 MΩ) of the cells obtained with our protocol were considerably lower than those of cultured primary PNs from acutely dissociated cerebella (54 ± 5 Hz and 900 ± 75 MΩ, respectively)^60^. Regarding spike activity, the difference may be partly accounted for by the considerably higher amount of synaptic input from granule cells received by primary PNs compared to NPC-derived PNs. Regarding the lower input resistance, the difference may be due to differences in the recording temperatures or the use of cesium in the recording solution^61^. We also investigated the functionality of the cells through electrophysiological means in an adapted 3D culture system, in which the NPCs were embedded in Matrigel^42^ and deposited in transparent µ-wells^43^. This combination allowed for spontaneous development of NPCs, including aggregation of calbindin-positive cells with spontaneous spike activity. The lower spiking frequency and membrane properties of PNs in the µ-wells as compared to those on the coverslips might be related to a different maturation level of the PNs cultured in the µ-wells^62–64^. The Matrigel-µ-well culture model proved especially useful for morphological reconstruction of the patched neurons, as it allowed intracellular filling of the PNs with biocytin. This was not possible with the coverslip approach because the neurons were more prone to bursting when biocytin was injected in the neurons on the coverslips than when biocytin was injected in the neurons embedded in Matrigel. Future cerebellar disease *in vitro* modeling should actively include 3D cell culture systems, which are increasingly important for culture of cells from patients^19, 65, 66^.

The maturation of NPCs *in vivo* was established via transplantation into the cerebellum of P1 or adult mice. Previous studies have shown the successful engraftment of ES-derived cells exclusively in embryonic and late postnatal cerebella^20, 52^. Here, we show maturation of ES-derived NPCs injected in not only early postnatal, but also adult, cerebella. Indeed ES-derived PNs could mature in the adult cerebellum of both wild-type mice and *L7-ERCC1* mutant mice that lack host Purkinje cells^27^. ES-derived PN progenitors and PN progenitors from the embryonic cerebellum show the correct integration when transplanted into the developing cerebellum^9, 17, 67^. Previous studies have reported that host and grafted Purkinje cells may compete to occupy the host molecular layer^4, 7, 15^, which could in principle lead to higher maturation of grafted PNs in a cerebellar environment depleted of PNs. However, our experiments did not show a better maturation in the *L7-ERCC1* mice, which suggests that other factors, such as those related to the intrinsic constitution of the grafted cells, also contribute.

The integration of transplanted NPCs obtained with the current protocol requires further work, because there was a low survival rate of injected cells while the vast majority of the grafted PNs occupied ectopic locations in the host molecular layer and showed no signs of cortico-nuclear projections. One of the significant challenges may be to circumvent endogenous cues, such as Reelin, in the adult cerebellar environment that are known to prevent the correct migration of cerebellar progenitors^17, 68, 69^. Indeed, when this challenge is not met, as in our current study, surviving grafted PNs will often remain displaced within the upper two-thirds of the host molecular layer and most of them will have inverted dendritic trees and local axonal terminal arborizations with varicosities^4, 5, 9, 15, 48^. In contrast to grafted cerebellar progenitors in developing cerebella, grafted cerebellar progenitors in the adult cerebellum do not migrate in high numbers to the Purkinje cell layer^17^ and do not provide prominent projections to the cerebellar nuclei^70, 71^, as also was the case in the current study. Possibly, both host and grafted Purkinje cells are particularly sensitive to toxic agents or critical environmental conditions^72^, because of their high intrinsic activity and metabolic demands^73^. Yet, when embryonic Purkinje cells are transplanted into an adult environment, they have in principle the ability to adopt several strategies to induce modifications in the host environment in order to create a permissive environment and make their way to the target position^48, 74^. Grafted embryonic PNs can induce modification in gene expression of the host glia in order to facilitate their radial migration in the adult molecular layer, which is called “adaptive rejuvenation”^74^. Similarly, migrating neurons can induce secretion of proteolytic enzymes so as to disrupt components of the host extracellular matrix, such as laminin of the basal lamina, and enter the host molecular layer from the interlobular space^48^. As a consequence, disruption of the recipient texture might even lead to displacement or removal of the host Purkinje cells. Moreover, because of this variety of interactive mechanisms, heterogeneous populations of cerebellar progenitors might be more efficient in inducing a successful transplantation than homogeneous subpopulations of Purkinje progenitors, as it increases the likelihood of containing the proper facilitating ingredients^20^.

As mentioned above, isolated PN progenitors in this study were not viable after sorting. The heterogeneous nature of the NPC population in our study may have had several consequences. For example, it may have facilitated the co-occurrence of calbindin-positive and calbindin-negative, GFP-labeled PCs following grafting. Our NPCs were probably subject to differential heterogeneous developmental events as opposed to PC progenitors derived from embryos, which traverse a homogeneous development with a well-synchronized expression of cellular markers. Conversely, the heterogeneity of the NPC population might also have protected some of the implanted PNs and facilitated the activation of endogenous compensatory functions in the recipient cerebellum^67^. This possibility is also in line with the fact that we found no evidence for fusion of grafted and host cells. Indeed, triple positive GFP-calbindin-mCherry cells were not observed following grafting in the cerebella of mCherry-knockin mice, nor were healthy resident Purkinje cells observed following grafting in the cerebella of *L7cre-ERCC1* knockout mice. Moreover, the morphology of the dendritic trees of differentiated GFP-positive-calbindin-positive PNs as observed 24 days after grafting was relatively immature as compared to host controls, highlighting the divergent developmental route. Likewise, different from embryonic cerebellar transplants performed with progenitors that have been exposed to the presence of afferences^48^, ES-derived NPCs are not exposed to an endogenous environment until grafting. As a consequence, a maturation period of 24 to 30 days may also be too short for ES-derived PCs to acquire the full extent of segregation of afferent inputs, including not only those of interneurons but also of climbing fibers. Thus, studies with NPCs maturing for longer periods of time are probably needed to evaluate both the development and maturation of ES derived NPCs and the formation of stable connections in the mouse cerebellum. Future studies will be required to resolve the extent to which proteolytic enzymes are responsible for displaced resident Purkinje cells^48, 74^ and/or injection-induced local hypoxia^75^ and to what extent progenitors and host cerebellum can be manipulated to enhance efficiency of integration. The occurrence of displaced or malformed Purkinje cells by itself may not necessarily have unavoidable negative consequences for possible therapeutic use. If the transplantation of ES-derived PC progenitors could replace missing PCs in human ataxia and if their synaptic inputs and outputs integrate in the cortical and downstream circuits, the secondary pathology resulting from the grafting process might have only a relatively minor functional impact. Hence, provided we can improve survival, migration and integration of NPCs in the host cerebellum, and limit the adverse impact of the grafting procedure, ES-derived NPCs might be considered as a source for human therapy in the future.

## Materials and Methods

### Animals

All experimental procedures involving animals were approved a priori by an independent animal ethical committee (DEC consult, Soest, The Netherlands) and/or by the national authority (*Centrale Commissie Dierproeven*, The Hague, The Netherlands) and performed in accordance to Dutch legislation and institutional guidelines (Erasmus MC, Rotterdam, The Netherlands and Netherlands Institute for Neuroscience, Amsterdam, The Netherlands).

Cerebellar differentiation protocols were performed on: i) actin-GFP ES cells derived from embryos obtained from crossings between a female wildtype 129 Sv mouse and a male heterozygous actin-GFP mouse^35^ with a C57BL/6 background; and ii) wild type ES cells derived from embryos obtained from crossings between female and male C57BL/6 mice. Injections in the adult cerebella involved wild types obtained from a cross between female and male C57BL/6, Purkinje-neuron-specific *L7cre-ERCC1* homozygous knockouts^27^ and CDK9-mCherry knockin^50^ mice with a C57BL/6 background.

### ES derivation and culture conditions

The ovarian cycle of female mice was synchronized by intra-peritoneal injections of pregnant mare serum gonadotrophin and human chorionic gonadotropin (both 150 µl, 50 U/ml, both one injection with 48 hrs interval). Following the last injection, each female mouse was mated with a male mouse. Females that were found with a vaginal plug ~20 hrs following the start of mating were euthanized at E3.5 and their embryos were collected. For ES derivation^31^, E3.5 blastocysts of actin-GFP mice and wild type mice were placed into culture dishes coated with gelatin (0.2%) and irradiated mouse embryonic fibroblasts (MEFs) in ES cell medium containing DMEM, 15% fetal calf serum (FCS, Lonza), PenStrep (PS, Sigma-Aldrich), 1 mM non-essential amino acids (NEAA, Sigma-Aldrich), 50 nM β-mercaptoethanol (Sigma-Aldrich), leukaemia inhibitory factor (LIF, Sigma-Aldrich), MEK inhibitor (4 µM, Stem cell technologies) and GSK3 inhibitor (3.3 µM, Stem cell technologies). One week after collecting embryos, the outgrowth of the inner cell mass was enzymatically split and plated by returning them to the same, but refreshed, culture conditions. After 3 passages, inhibitors for MEK and GSK3 were removed from the culture medium.

### ES cell culture

ES cells were maintained as described^28^ for up to 6 passages before differentiation: starting with 2 passages on inactivated MEFs followed by at least 2, but not more than 4, passages on gelatine-coated (0.2% gelatine solution in water) T-25 flasks (Nunc, Denmark). ES culture medium contained DMEM (Gibco) supplemented with 15% FCS (Lonza), 1000 U/ml leukaemia inhibitory factor (LIF, Sigma-Aldrich), 1% non-essential amino acids (Sigma-Aldrich), 0.1 µM β-Mercaptoethanol (Sigma-Aldrich). Cells were split at 1:4 at every passage.

### ES cell embryoid body (EB) formation and differentiation

ES cells were dissociated in 0.25% trypsin-EDTA (Sigma-Aldrich) and reaggregated in 96-well low-cell-adhesion plates (Lipidure coat, NOF, Japan) at seeding densities of 3 × 10^3^ and 3 × 10^4^ cells per 150 µl of medium to induce cerebellar differentiation of mouse ES cells in serum-free culture of embryoid-body like aggregates (SFEBq)^20^. The differentiation medium contained: Isocove’s modified Dulbecco’s medium (Gibco)/Ham’s F12 (Gibco) 1:1, chemically defined lipid concentrate (1% of total volume, Sigma-Aldrich), penicillin/streptomycin (Sigma-Aldrich), monothioglycerol (450 µM, Sigma-Aldrich), human apo-transferrin (15 µg/ml, Sigma-Aldrich), insulin (7 µg/ml, Sigma-Aldrich) and crystallization-purified BSA (5 mg/ml, Sigma-Aldrich). In addition, 4 × 10^6^ ES cells^28^ were seeded per Petri dish (Greiner Bio-one) in differentiation medium.

### Neuronal differentiation culture

Unless stated otherwise, FGF2 (20 ng/ml, Life Technologies) and Cyclopamine (10 µM, Bio-connect, The Netherlands) were added to SFEBq cultures on days 1 and 7, respectively^20^. SFEBq and petri dish (Greiner Bio-One) cultures were placed in an incubator (Heraeus, Germany) for up to 17 days under static conditions and/or dynamic conditions (100 RPM, IKA Vibrax VXR, Germany), as indicated.

### Neural progenitor cell (NPC) culture

EBs in differentiation medium were collected from 9 to 17 days *in vitro* (DIV) in a 15 ml tube. Then EBs were centrifuged at 300 g for 5 min, differentiation medium was removed and EBs were re-suspended in NPC medium containing: DMEM/F12 (Gibco), N2 supplement (1%, Invitrogen), B27-RA supplement (1:50, Invitrogen), laminin (1 µg/ml, Sigma-Aldrich), mouse bFGF (20 ng/ml, Invitrogen), penicillin/streptomycin (1%, Sigma-Aldrich). EBs re-suspended in NPC medium from one 96 well plate were split into two 10 cm^2^ wells of 6-well culture dishes (Greiner Bio-One), which were coated with laminin (50 ug/ml, Sigma-Aldrich) for at least 30 min at 37 °C. NPCs were seeded immediately after removing excess of laminin. Adherent cultures were refreshed every other day with NPC medium and passaged (1:4) every 4 days for up to 6 passages. NPCs could be preserved at every passage after washing with PBS, detaching with 0.25% trypsin-EDTA (Sigma-Aldrich) for 5 min at 37 °C, resuspension in DMEM/F12 (Gibco) with 10% FBS (Lonza), centrifugation at 300 g for 5 min, followed by resuspension of NPCs in FBS containing 10% DMSO (Sigma-Aldrich) in cryotubes.

### Cerebellar differentiation of NPCs

NPCs were detached from 10 cm^2^ wells as mentioned above and resuspended in NS21 medium^26^ containing primary neural basal medium (PNBM, Lonza), GS21 (2%, GlobalStem), glutamax (1%, Gibco) and gentamycin (0.5 µg/ml). A 300 µl drop of NPCs suspension was seeded on 13 mm coverslips (Assistent, Germany) previously coated overnight at RT with polyornithine (0.5 mg/ml, Sigma-Aldrich) followed by at least 30 min coating with laminin (50 µg/ml, Sigma-Aldrich) at 37 °C. The volume of non-attached cells was removed after 1 hr and NS21 medium (600 µl) was added to the culture (0 DIV). Next, neurons were refreshed (600 µl) with NS21 medium supplemented with cytosine β-D-arabinofuranoside (4 µM, Ara-C, Sigma-Aldrich) every 48 hrs.

For 3D culture, µ-wells (3 µl per µ-well, Vers3D^TM^, Screvo B.V., The Netherlands)^43^ made of Epoxy (Epotek stycast 1264 A/B, Epoxy Technology, Belgium) were seeded with 3 µl per µ-well of a cell suspension consisting of NPCs in NS21 diluted in Matrigel (1:2, BD Biosciences)^42^. The final cell concentration in the mixture was ≥1 × 10^7^ cells/ml. The cell/Matrigel mixtures in the µ-wells were incubated at 37 °C for 5 min before 1 ml of pre-warmed NS21 was added to completely cover each µ-well in 24-well plates.

### Flow cytometric analyses of cell aggregates

Cells were sorted using a BD FACSARIA III (BD Biosciences, San Jose, CA). Single-cell suspensions of EBs in 96-well plates were obtained by collecting all EBs of one 96-well plate in one 15 ml tube, centrifugation at 1500 RPM for 2 min, followed by removal of 14 ml of supernatant. Then, 0.5 ml of Accumax (Chemicon, Germany) was added to the EBs in 0.5 ml of medium and the contents were mixed with a 1 ml pipette and incubated for 15 min at room temperature. For Neph3-positive cells isolation and measurement, EBs were dissociated with Accumax as described above on 11, 13, 15 and 17 DIV and filtered through a cell strainer (BD Biosciences). Isolated cells were incubated in Neph3 monoclonal antibody (30 min, Sheep 1:200, R&D Systems) and labeled with a PE-conjugated secondary antibody (30 min, BD Biosciences). Doublets were excluded from the analysis. Sorted cells were gathered in ice-cold DMEM/F12/N2/10%FCS or NPCs medium and aggregated again in low-cell adhesion 96-well culture plates or 24-well plates.

### Electrophysiology

Cells cultured on coverslips were recorded at room temperature (21 ± 2 °C), during which the extracellular bath solution contained (in mM): 124 NaCl, 2.5 KCl, 1.25 Na_2_HPO_4_, 2 MgSO_4_, 2 CaCl_2_, 26 NaHCO_3_ and 20 D-Glucose (osmolarity: 295 Osm/L). Because NS21 medium has a lower osmolarity (255 Osm/L) than the extracellular bath solution, cultured neurons were gradually brought to equilibrium with the solutions before recording. This was done by adding 200 µL of extracellular solution to the cultured neurons in NS21 medium and removing 200 µL of the mixed solution every 3 minutes for a total of 20 min. Cell-attached and patch-clamp recordings were recorded in the same session. Electrodes with resistances of 4 to 8 MΩ were pulled from thick-walled borosilicate glass capillaries and filled with (in mM): 9 KCl, 10 KOH, 3.48 MGCl_2_, 4 NaCl, 120 K-Gluconate, 10 Hepes, 28.5 Sucrose, 4 Na_2_ATP and 0.4 Na_3_GTP. An Axopatch 700B amplifier (Molecular Devices) was used for recordings. Data were filtered at 1 kHz using pClamp software and with offline analysis performed with Clampfit 10.5 (Molecular Devices). All recordings were performed between 13 and 18 DIV.

Neurons were recorded in whole-cell configuration with the same solutions, pipettes, equipment and analysis software as described above. Membrane test settings were recorded in voltage clamp with the holding potential at −65 mV. We recorded the resting membrane potential in current clamp without a bias current directly after breaking the giga-seal. This potential was corrected for the liquid junction potential (−10.5 mV).

### NPC injections in early postnatal and adult cerebella

Cell injections were performed on eight P1 mice. P1 mice were cryo-anesthetized, and the cerebellum was localized by trans-illumination of the head. A single cell suspension of NPCs, at a concentration of 5 × 10^4^ cells/µl in NS21 medium and labeled with Fast Green, was injected by means of a glass micropipette with a tip diameter of around 60–80 µm and a pneumatic pressure injection apparatus (Picospritzer II, General Valve, Fairfield). About 1 µl of cell suspension was administered at each injection site, which included the vermis and each hemisphere of the cerebellum.

Eight 4-month-old and eight 10-month-old wild type animals from different litters, three 5-month-old cyclin-dependent-kinase-9 (CDK9)-mCherry knockin^50^ heterozygous mice and three 8 month old littermates *L7cre-ERCC1* ^27^ homozygous mice were anesthetized through inhalation of 5% isoflurane in O_2_ for 2 hrs. A single cell suspension at a concentration of 5 × 10^4^ cells/µl in NS21 medium was obtained from cultured NPCs. Wild type and homozygous L7Cre-ERCC1 KO mice received three injections (1.5 µl total volume at 0.5 µl/min) of the final cell suspension using a stereotaxic apparatus with a Hamilton syringe (Hamilton, Germany) according to the following coordinates from Lambda targeting the deep cerebellar nuclei (DCN), lobule 4/5, simple lobule and crus1 of the cerebellum, respectively: (a) AP −2.4 mm; ML −2.0 mm; DV 2.0 mm b) AP −2.4 mm; ML 0 mm; DV 1.0 mm c) AP −2.4 mm; ML 1.5 mm; DV 1.2 mm d) AP −2.4 mm; ML 2.7; DV 1.0.

### Immunohistochemistry

EBs were collected from 11 to 15 DIV in 96-well plates and concentrated in 50 ml tubes. Subsequently these were centrifuged at 300 g for 5 min and the supernatant was removed. EBs were then re-suspended in paraformaldehyde (PFA, 4%), transferred to a 1.5 ml tube and stored overnight at 4 °C. EBs in 1.5 ml tubes were washed twice with phosphate buffer after centrifugation, followed by careful removal of the supernatant. Immediately after EBs were embedded in Tissue-Tek (Sakura Finetek Europe, The Netherlands) and stored at −80 °C. Sections (10 µm) of EBs were obtained using a Cryotome (Microm HM 560, Microm Gmbh, Germany) stained on glass overnight at 4 °C with primary antibodies and for 2 h with fluorescent secondary antibodies (see below for antibodies and dilutions).

Neurons on coverslips were fixed (4% PFA) for 15 min at 4 °C and treated with primary and secondary antibodies as described above. Neurons/Matrigel in µ-wells were incubated for 2 nights at 4 °C with primary antibodies and overnight at RT with fluorescent secondary antibodies. To prevent the Matrigel from disintegrating µ-well antibody stainings were performed in a solution containing: 0.05 M Tris buffer, 0.9% NaCl, 0.25% gelatine, and 0.5% Triton-X-100 at pH 4. For visualization of recorded neurons, biocytin was added to the intracellular solution, incubated in 4% PFA for two nights and treated with streptavidin-Cy3.

Free floating sections were processed with antibodies as described above for EBs sections. For quantitative analyses, at least 3 images from over 3 experiments were evaluated. The following antibodies were used at the described dilutions: Calbindin (Mouse 1:7000, Sigma-Aldrich, Rabbit 1:10000 Swant), E-cadherin (Mouse 1:200, Abcam), En2 (Goat 1:50, Santa Cruz), GFAP (Mouse 1:15000, Sigma-Aldrich, Rabbit 1:8000, Dako), Neph3 (Sheep 1:200, R&D Systems), NeuN (Mouse 1:2000, Chemicon), Parvalbumin (Mouse 1:7000 Swant, Rabbit 1:7000 Swant), VGlut1 (Rabbit 1:1000 Synaptic Systems), VGlut2 (Guinea pig 1:2000 Millipore), SMI32 (Mouse 1:5000 Sternberger). DAPI (Molecular Probes) was used as a nuclear counterstain. Cy3 (mouse/rabbit/goat 1:200, Dako), and Cy5 (mouse/rabbit/sheep 1:200, Dako) were used as secondary antibodies. Images were obtained on an upright LSM 700 confocal microscope (Zeiss), evaluated with FIJI^76^ and assembled by Adobe illustrator CS6.

### Statistical Analysis

The mean and deviation of measurements were evaluated in Matlab (Mathworks) using one-way ANOVAs followed by Tukey’s post-hoc testing with statistical significance defined at p < 0.01, unless stated otherwise. All data are reported as mean ± standard error of measurements (SEM).

## Electronic supplementary material

Supplementary Information
