# Supplementary material for: An expandable embryonic stem cell-derived Purkinje neuron progenitor population that exhibits in vivo maturation in the adult mouse cerebellum
Source: Sci Rep. 2017 Aug 18;7:8863. doi: 10.1038/s41598-017-09348-1 (PMC5562837; doi:10.1038/s41598-017-09348-1)
Supplement: Supplementary file 1 — Supplementary Information [file 41598_2017_9348_MOESM1_ESM.pdf]

Supplementary Information File

An expandable embryonic stem cell-derived Purkinje neuron progenitor population that exhibits *in vivo* maturation in the adult mouse cerebellum

Gustavo A. Higuera*[1], Grazia Iaffaldano*[1,2], Meiwand Bedar[1]*, Guy Shpak[4], Robin Broersen[1,2], Shashini T. Munshi[4], Catherine Dupont[3], Joost Gribnau[3], Femke M.S. de Vrij[4], Steven A. Kushner[4] and Chris I. De Zeeuw[1,2]

[1]Department of Neuroscience, Erasmus MC Rotterdam, NL-3015 GE Rotterdam, The Netherlands

[2]The Netherlands Institute for Neuroscience, Royal Netherlands Academy of Arts and Sciences, 1105 BA Amsterdam, The Netherlands

[3]Department of Endocrinology & Reproduction, Erasmus MC Rotterdam, NL-3015 GE Rotterdam, The Netherlands

[4]Department of Psychiatry, Erasmus MC Rotterdam, NL-3015 GE Rotterdam, The Netherlands

*These authors contributed equally to this work.

Correspondence: g.higuerasierra@erasmusmc.nl or c.dezeeuw@erasmusmc.nl

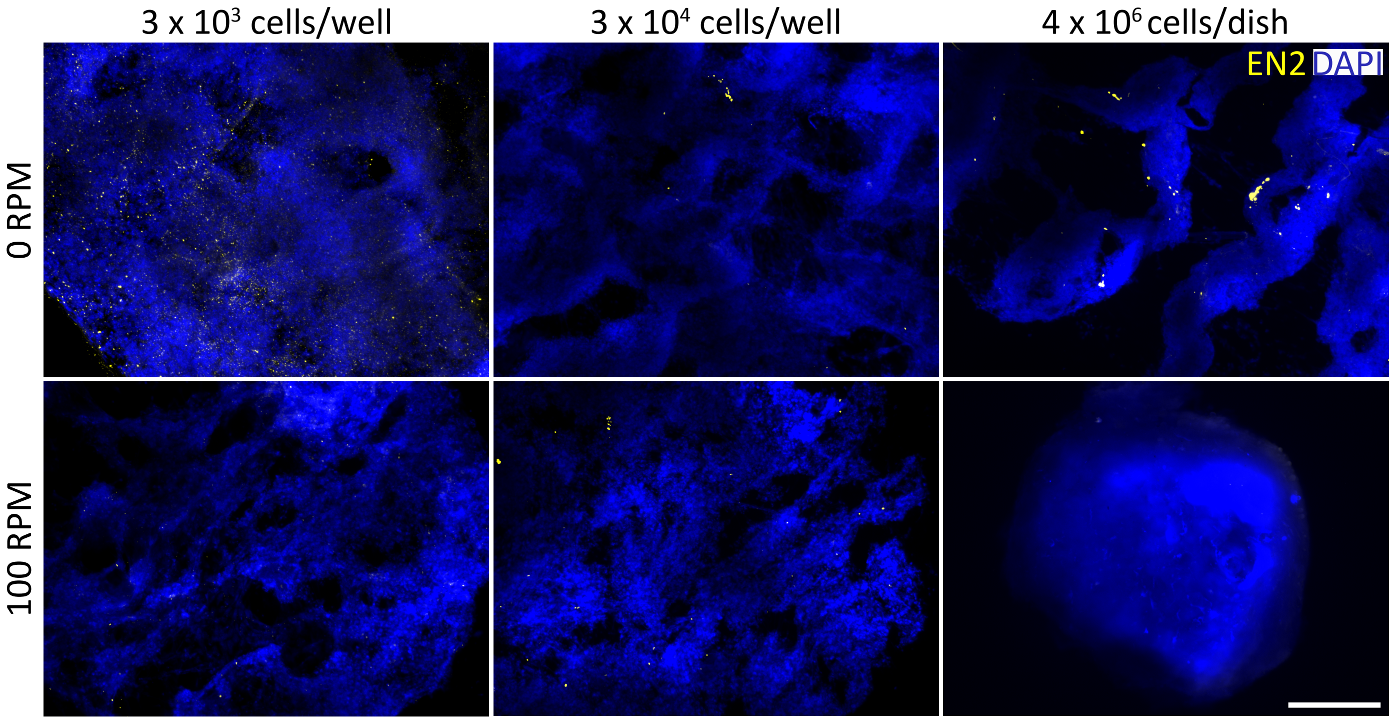

Supplementary figure 1. Midbrain-hindbrain boundary (MHB) formation in EBs with different cell densities in differentiation medium with cyclopamine after 8 days in vitro (DIV) under static (obtained at 0 RPM) or dynamic (obtained at 100 RPM) conditions. Expression of Engrailed2 is highest under static conditions (i.e. top panel on the left). Scale bar indicates 200 µm.

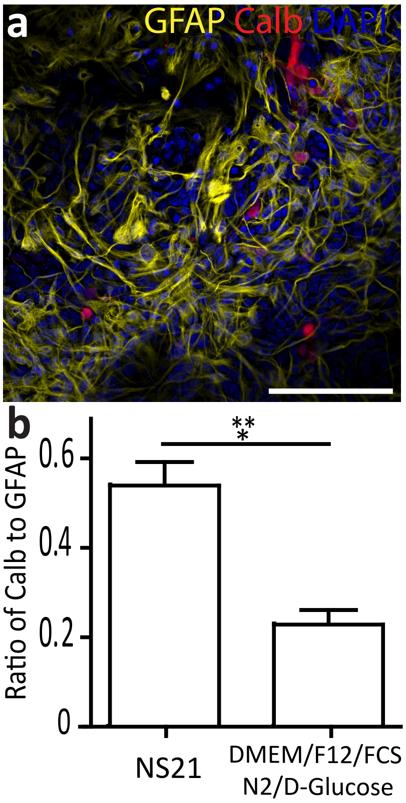

Supplementary figure 2. Cell culture media comparison. (a) Culture of NPCs in DMEM/F12/FCS/N2/D-Glucose medium from Muguruma et al. (2010)20. For NPCs cultured in NS21 media, see main Figure 4. (b) Ratio of calbindin-positive to GFAP-positive cells in NS21 medium vs. DMEM/F12/FCS/N2/D-Glucose medium. The ratio of calbindin-positive cells to GFAP-positive was significantly higher ($p < 0.001$) in NS21 media. *P<0.01, **P<0.001.

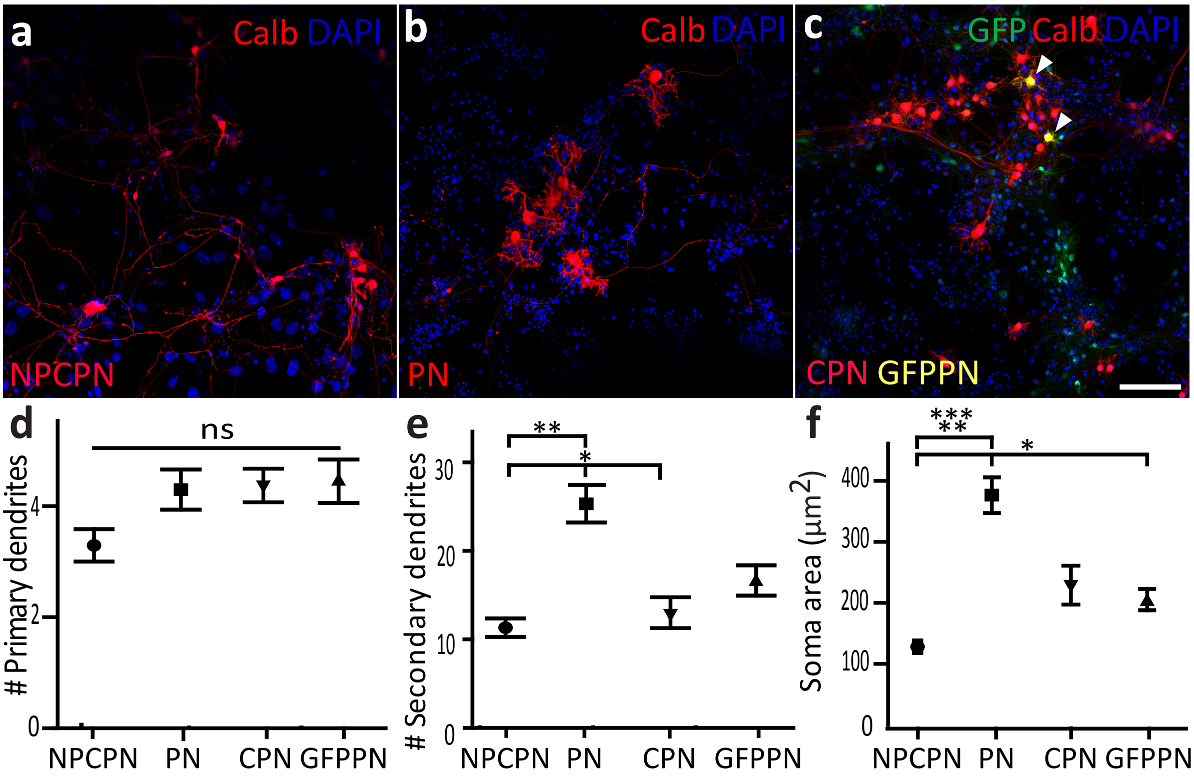

Supplementary figure 3. PNs maturation in NS21 medium either as separate dissociated cultures (PN) or in co-culture with NPC derived PNs (NPCPN), co-cultures of dissociated PNs (CPN) or NPCs (GFPPN) (a-c). The arrowheads in panel c indicate actin-GFP-positive calbindin-positive NPC-derived cells. Scale bars: 100 µm (a-c). Quantification of (d) primary dendrites, (e) secondary dendrites, and (f) soma area (n = 20 neurons/condition). Charts show the mean and s.e.m. (n=10 images from 3 experimental runs involving 3 batches of cells) with statistical comparisons assessed via one-way Anova followed by Tukey's with statistical significance set at $p \leq 0.01$. *P<0.01, **P<0.001, and ***P<0.0001.

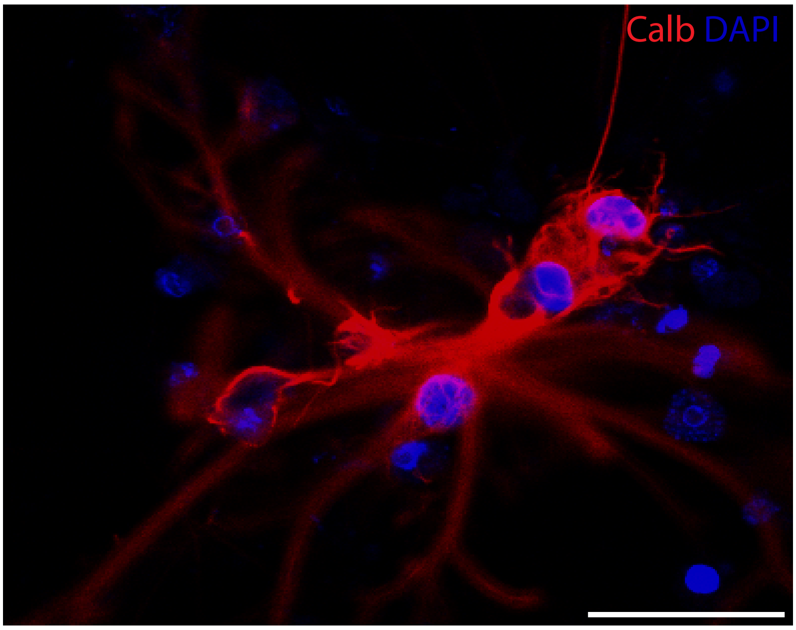

Supplementary figure 4. EBs can be directly transferred from the differentiation stage to the maturation stage in NS21 medium, resulting at 30 DIV in calbindin-positive cells with a dendritic tree. Scale bar: 100 µm.

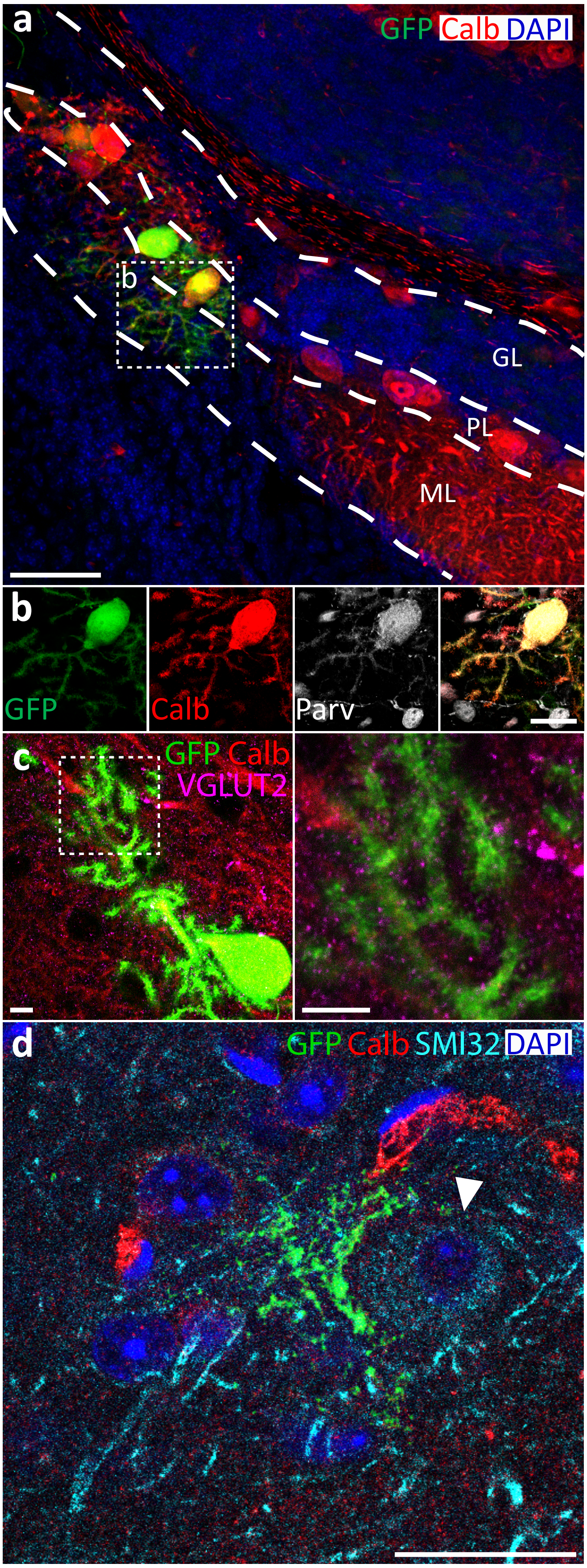

Supplementary figure 5. NPCs morphologically matured into PNs and integrated in the postnatal (P1) mouse cerebellum 30 days after grafting. (a-b) GFP-positive PNs migrated to the Purkinje cell layer, developed dendrites with spines in the right orientation and co-expressed calbindin and parvalbumin. (c) Glutamate transporter VGluT2 puncta were detected on the dendritic shafts of GFP-expressing neurons. (d) Actin-GFP-positive axons terminate at the soma of an SMI-32-positive glutamatergic neuron in the cerebellar nuclei. Arrowhead points to the soma of the cerebellar nuclei neuron. Scale bars: 20 µm (a, b and d); and 5 µm (c). *all micrographs represent coronal sections.

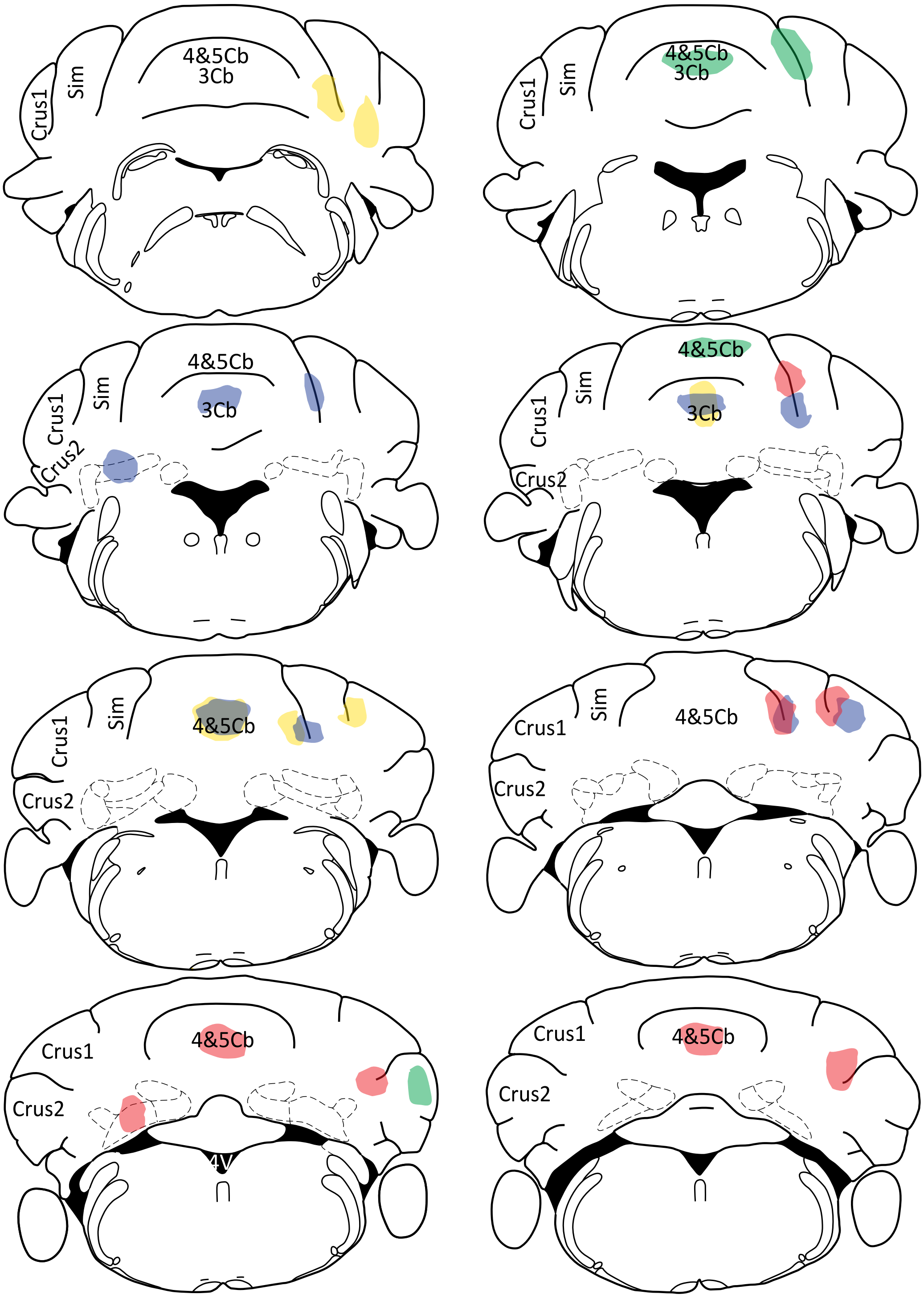

Supplementary figure 6. Eight serial coronal sections of the mouse cerebellum showing the distribution of injected NPCs in 4 cerebella (as indicated by distinct colors), which are representative examples of the cerebellar position of the injections in 16 adult wild-type (4- and 10-month old), 3 L7-Cre ERCC1, and 3 CDK9-mCherry mice. Serial sections were obtained from Paxinos and Franklin (2001)[2], (left to right = rostral to caudal) and the anatomical position of injections was drawn on top of them. 4&5Cb: lobule 4/5, 3cb: lobule 3, sim: simple lobule. This figure is not covered by the CC BY licence. [© Elsevier Inc.]. All rights reserved. Used with permission.

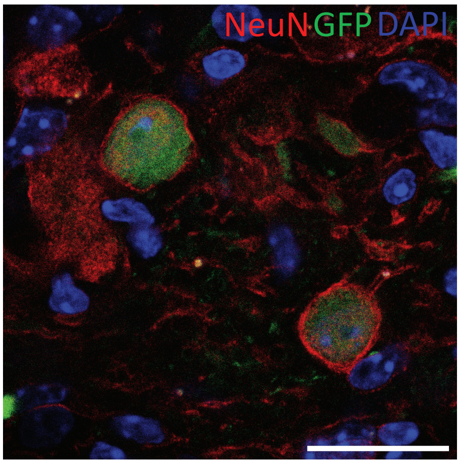

Supplementary figure 7. GFP-positive cells 30 days after grafting in a 10-month old adult mouse cerebellum with a developed dendritic tree and NeuN immunoreactivity, highlighting the interneuron identity. Scale bar: 40 µm.

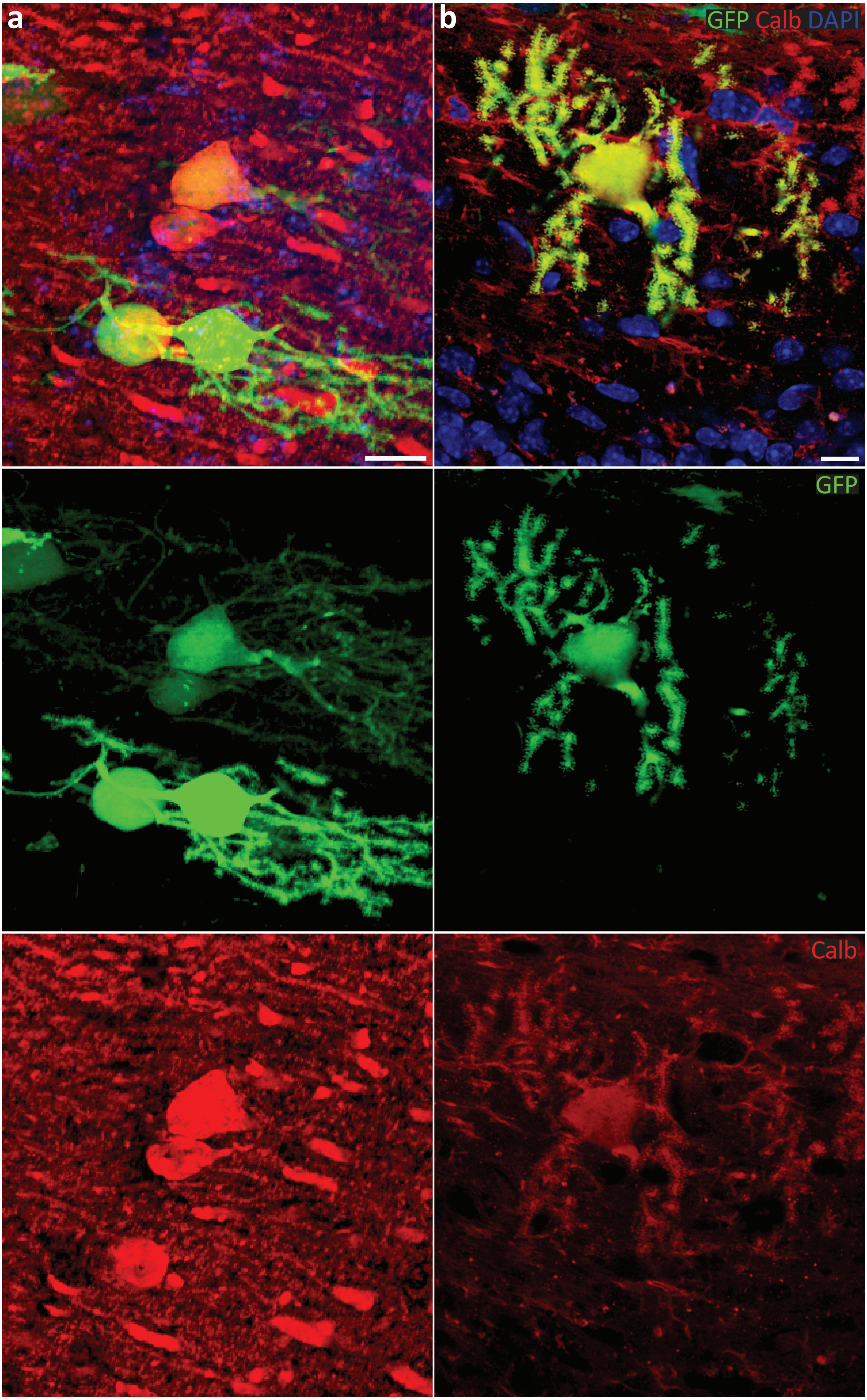

Supplementary figure 8. Thirty days after transplantation into the adult (10-month old) wild type cerebellum some of the donor GFP-positive cells differentiated into Purkinje cells as shown by the typical dendritic tree characterized by the presence of the spines and calbindin. (a and b) Various examples showing GFP-positive-calbindin-positive cells. Scale bars: 20 µm (a); 10 µm (b). *all micrographs represent coronal sections.

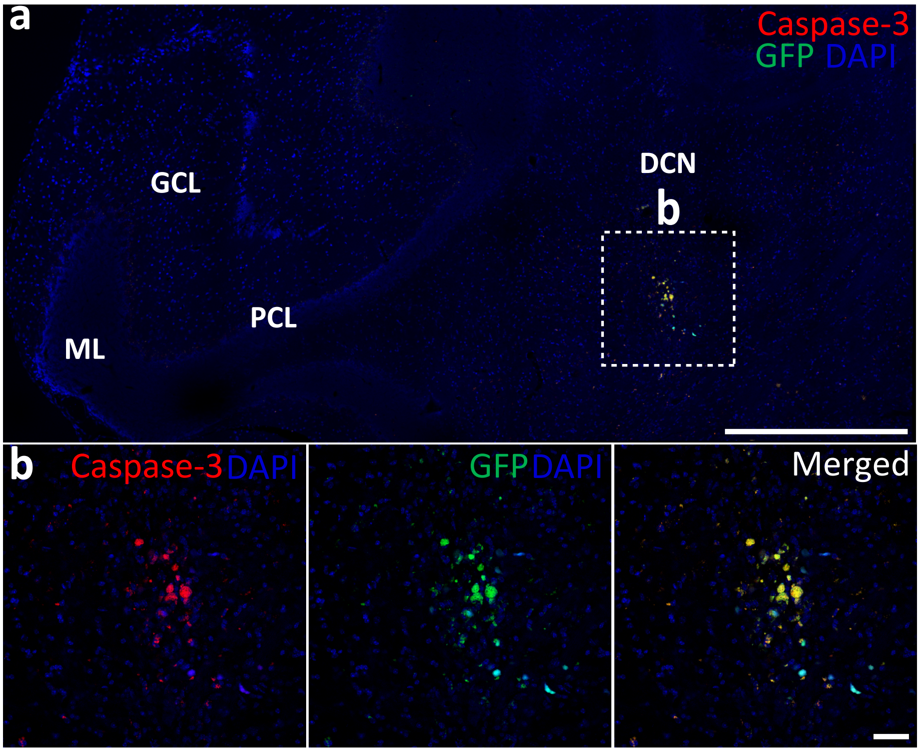

Supplementary figure 9. NPC injections in the cerebellar nuclei of adult (4-month old) wild type mice led to cell death (a), as displayed by caspase-3 labeling (b). Scale bars: 500 µm (a); and 40 µm (b). ML: Molecular layer; PCL: Purkinje cell layer; GCL: Granule cell layer. Micrographs were obtained on sagittal sections 30 days after grafting.

References

1. Muguruma, K., Nishiyama, A., Ono, Y., Miyawaki, H., Mizuhara, E., Hori, S., Kakizuka, A., Obata, K., Yanagawa, Y., Hirano, T. & Sasai, Y. Ontogeny-recapitulating generation and tissue integration of ES cell-derived Purkinje cells. Nature Neurosci. 13, 1171-1180; DOI: 10.1038/nn.2638 (2010).

2. Paxinos, G. & Franklin, K. B. J. The Mouse Brain in Stereotaxic Coordinates. Second edn, 296 (Academic Press, 2001).
